# Bioactivity-driven fungal metabologenomics identifies antiproliferative stemphone analogs and their biosynthetic gene cluster

**DOI:** 10.1007/s11306-024-02153-8

**Published:** 2024-08-02

**Authors:** Navid J. Ayon, Cody E. Earp, Raveena Gupta, Fatma A. Butun, Ashley E. Clements, Alexa G. Lee, David Dainko, Matthew T. Robey, Manead Khin, Lina Mardiana, Alexandra Longcake, Manuel Rangel-Grimaldo, Michael J. Hall, Michael R. Probert, Joanna E. Burdette, Nancy P. Keller, Huzefa A. Raja, Nicholas H. Oberlies, Neil L. Kelleher, Lindsay K. Caesar

**Affiliations:** 1 Department of Chemistry, Northwestern University, Evanston, IL USA; 2 Proteomics Center of Excellence, Northwestern University, Evanston, IL USA; 3 Department of Chemistry and Biochemistry, University of North Carolina at Greensboro, Greensboro, NC USA; 4 Department of Chemistry and Biochemistry, James Madison University, Harrisonburg, VA USA; 5 Department of Molecular Biosciences, Northwestern University, Evanston, IL USA; 6 College of Pharmacy–Pharmaceutical Science, University of Illinois Chicago, Chicago, IL USA; 7 Chemistry, School of Natural and Environmental Sciences, Newcastle University, Newcastle Upon Tyne, NE1 7RU UK; 8 Department of Chemistry, Universitas Indonesia, Depok, Jawa Barat Indonesia; 9 Indicatrix Crystallography, Newcastle University, Newcastle Upon Tyne, NE1 7RU UK; 10 Department of Medical Microbiology and Immunology, University of Wisconsin-Madison, Madison, WI USA; 11 Department of Bacteriology, University of Wisconsin-Madison, Madison, WI USA

**Keywords:** Natural products, Metabologenomics, Biosynthesis, Biochemometrics, Fungi, Secondary metabolism

## Abstract

**Introduction:**

Fungi biosynthesize chemically diverse secondary metabolites with a wide range of biological activities. Natural product scientists have increasingly turned towards bioinformatics approaches, combining metabolomics and genomics to target secondary metabolites and their biosynthetic machinery. We recently applied an integrated metabologenomics workflow to 110 fungi and identified more than 230 high-confidence linkages between metabolites and their biosynthetic pathways.

**Objectives:**

To prioritize the discovery of bioactive natural products and their biosynthetic pathways from these hundreds of high-confidence linkages, we developed a bioactivity-driven metabologenomics workflow combining quantitative chemical information, antiproliferative bioactivity data, and genome sequences.

**Methods:**

The 110 fungi from our metabologenomics study were tested against multiple cancer cell lines to identify which strains produced antiproliferative natural products. Three strains were selected for further study, fractionated using flash chromatography, and subjected to an additional round of bioactivity testing and mass spectral analysis. Data were overlaid using biochemometrics analysis to predict active constituents early in the fractionation process following which their biosynthetic pathways were identified using metabologenomics.

**Results:**

We isolated three new-to-nature stemphone analogs, 19-acetylstemphones G (**1**), B (**2**) and E (**3**), that demonstrated antiproliferative activity ranging from 3 to 5 µM against human melanoma (MDA-MB-435) and ovarian cancer (OVACR3) cells. We proposed a rational biosynthetic pathway for these compounds, highlighting the potential of using bioactivity as a filter for the analysis of integrated—Omics datasets.

**Conclusions:**

This work demonstrates how the incorporation of biochemometrics as a third dimension into the metabologenomics workflow can identify bioactive metabolites and link them to their biosynthetic machinery.

**Supplementary Information:**

The online version contains supplementary material available at 10.1007/s11306-024-02153-8.

## Introduction

Secondary metabolites (natural products) from bacteria, fungi, and plants have had major impacts on human society, producing many commercially-used small molecule pharmaceuticals and agrochemicals. Fungi biosynthesize a plethora of chemically and structurally diverse secondary metabolites (Macheleidt et al., 2016), many of which have found use as drugs, pigments, dyes, antioxidants, agrochemicals, and other consumer products (Bills & Gloer, 2016). Despite the hundreds of fungal secondary metabolites that have found use in human society, it is estimated that less than 7% of the 5 million fungal species on the planet have been studied for bioactivity (Blackwell, 2011; Wu et al., 2019), highlighting the untapped nature of the fungal kingdom for the discovery of new natural products.

Most biosynthetic pathways required for secondary metabolite synthesis are organized in a contiguous fashion as biosynthetic gene clusters (BGCs) (Keller, 2019) that evolve by various genetic events including gene duplication, fusion, deletion, transposition, neofunctionalism, functional divergence, horizontal or lateral gene transfer, and de novo assembly (Rokas et al., 2018). Hence, studying fungal biosynthesis can aid in understanding fungal evolution, phylogeny, and ecological niche (Gupta et al., 2022; Kohler et al., 2015; Miyauchi et al., 2020; Wang et al., 2018). Moreover, knowledge of fungal biosynthetic and transcriptional machinery can enable genetic manipulation and enhanced production of secondary metabolites (Bok et al., 2006; Fox & Howlett, 2008).

Despite the promise of fungal natural products and the benefits of understanding their biosynthesis, the majority of fungal metabolites have yet to be linked to their biosynthetic pathways. In a prior study, we demonstrated that roughly 37,000 BGCs identified from 1037 publicly available fungal genomes have unknown metabolite products (Robey et al., 2021). In recent years,—Omics technologies (genomics, metabolomics, and integrated analyses) have become popular tools to access unexplored natural products space. The integrated ‘metabologenomics’ approach enables the association of a metabolite to its BGC by feature-based or correlation-based computation and comparison of the metabolomic and genomic data (van der Hooft et al., 2020). While numerous studies have made use of paired metabolomics and genomics datasets for discovery of bacterial natural products and their biosynthetic pathways (Caesar et al., 2021), this strategy has only recently been applied to higher organisms such as fungi. In a previous study by our team, we correlated genomics and metabolomics datasets from 110 fungi, delineating the biosynthetic pathway for the pestalamides (Caesar et al., 2023). This study also revealed 238 high-confidence natural product-BGC pairs for future study.

While metabologenomics has shown success for linking metabolites to their biosynthetic machinery, it only allows inferences about the biological activity of detected metabolites. A major bottleneck of natural products research is metabolite purification, and methods to prioritize biologically active metabolites are needed. In recent years, a multivariate statistical approach termed ‘biochemometrics’ has been used to integrate quantitative chemical information such as liquid chromatography-mass spectrometry (LC–MS) data and biological activity data into a statistical model to help discover chemical patterns related to bioactivity (Kellogg et al., 2016). These features can be prioritized for targeted study early in the fractionation process while also avoiding re-discovery associated with traditional bioactivity-guided fractionation.

**Fig. 1:**
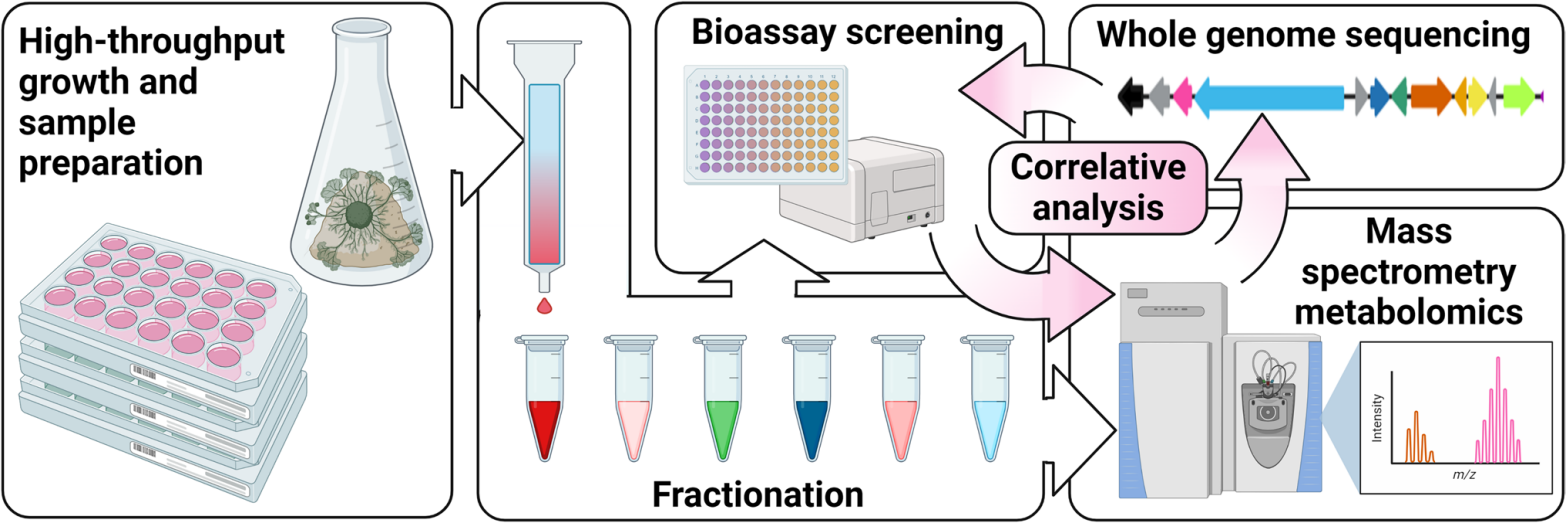
Bioactivity-guided metabologenomics workflow for prioritizing bioactive natural product-BGC pairs for targeted downstream analyses

In this work, we demonstrate integration of bioactivity into our metabologenomics workflow (Fig. 1). Using our previously published dataset of 110 Ascomycetes, combined with high-throughput antiproliferative bioactivity data, we assessed the ability of bioactivity-driven metabologenomics to prioritize biologically active metabolite-BGC pairs for downstream analyses. Using this platform, we targeted and isolated three new endogenous stemphone analogs, confirmed their antiproliferative activity against human melanoma and ovarian cancer cell lines, and proposed a rational biosynthesis for their production. This work highlights the promise of using biological activity as a filter for unwieldy integrated—Omics datasets, enabling prioritized study of bioactive metabolites and their biosynthetic machinery.

## Materials and methods

The detailed protocols are described in Supplementary Materials and Methods.

### Fungal growth and metabolome extraction

Fungal collection, growth, and secondary metabolite extraction were conducted as previously described (Caesar et al., 2023). Briefly, fungal strains were inoculated in Erlenmeyer flasks containing autoclaved rice, oats, or Cheerios and incubated for 2–5 weeks. Secondary metabolite extraction was then completed using a 1:1 mixture of chloroform: methanol, followed by filtration, liquid–liquid partitioning, and sample preparation for LC–MS/MS analysis and bioactivity screening (Al Subeh et al., 2021; Graf et al., 2020).

### Liquid chromatography tandem high-resolution mass spectrometry (LC-HRMS/MS) analysis for metabologenomics correlations

Dried fungal extracts at a concentration of 1 mg/mL were subjected to positive mode LC–MS/MS untargeted metabolomics analysis using a Thermo Q Exactive mass spectrometer coupled with an Agilent 1290 Infinity II ultrahigh performance liquid chromatograph. The chromatographic conditions included a binary solvent system, and MS parameters were set with a resolution of 17,500 for the mass range of 150–2000 *m/*z, utilizing data-dependent fragmentation analysis for MS^2^ data collection. Dereplication of known metabolites was conducted as described previously using extensive in-house dereplication databases (Caesar et al., 2023; El-Elimat et al., 2013; Paguigan et al., 2017). Feature detection and processing of LC–MS data were performed using MZmine v. 2.53 (Pluskal et al., 2010) as previously described (Caesar et al., 2023).

### Metabologenomics analysis

BGCs and gene cluster families (GCFs) were identified as previously described (Caesar et al., 2023). Binary correlations between subsets of each GCF based on clustered domain sequences present in each genome and the MS^1^ intact masses found in each extract were then calculated, following which scoring was conducted using three correlation-based metrics: pattern matching, correlation scoring, and intensity ratio analysis. All analyses were implemented in C#10 running on.NET 6 (Caesar et al., 2023).

### Primary high throughput screening assay for identifying bioactive fungal extracts

To prioritize from among over 200 significant natural product-BGC pairs identified previously (Caesar et al., 2023), fungal extracts were screened for bioactivity using a CellTiter-Glo® (Promega) luminescence assay against A549 (lung; XY genotype), HCT-116 (colon; XY genotype), LN-229 (brain/glioblastoma; XX genotype), and MCF7 (breast; XX genotype) cancer cell lines, and a normal cell line MCF-10A (breast; XX genotype). Extracts were added to cells at a final concentration of 50 µg/mL using an Echo 550 Acoustic liquid transfer system (Labcyte Inc.). Extracts showing antiproliferative activity were further tested at 25, 50 and 100 µg/mL to identify the most potent bioactive strains. Doxorubicin was used as a positive control at concentrations of 12.5, 25, and 50 µM.

### Fractionation and antiproliferative activity screening of bioactive fungal extracts

Out of 110 strains tested, 12 showed potent antiproliferative activity against our cell panel. Among these, three extracts (*Aspergillus biplanus* NRRL 5071, *A. diversus* NRRL 5074, and *A. conjunctus* NRRL 5080) with similar chemical and sensitivity profiles were selected and separated using flash chromatography. Extracts (ranging from 200 mg to 4 g) underwent normal-phase flash chromatography, and fractions were combined based on UV–Vis absorbance profiles for subsequent bioactivity testing and metabolomics analysis.

For biochemometrics correlations, flash chromatography fractions were tested against MCF7 cells with doxorubicin as positive control. Cells were grown in 96-well plates and incubated for 24 h before extracts were added in triplicate at final concentrations of 50 and 16.5 µg/mL. Following a 48-h treatment period, cell proliferation was evaluated using a 3-[4,5-dimethylthiazol-2-yl]-2,5 diphenyl tetrazolium bromide (MTT) assay, and absorbance at 600 nm was measured to determine cell viability.

### LC–HRMS/MS-based correlation of metabolomics and bioactivity datasets

LC–MS/MS spectra of flash chromatography fractions were acquired, and files were converted to .mzXML format using ProteoWizard (Chambers et al., 2012). Feature detection and processing of LC–MS data were performed using MZmine v. 2.53 (Pluskal et al., 2010) using modified parameters outlined in Table S1 to create a feature list table for biochemometrics correlations. Additionally, .mzXML files were uploaded to the Global Natural Products Social Molecular Networking (GNPS) site (Wang et al., 2016) to visualize molecular families based on specified criteria.

The resulting metabolite feature table was merged with the bioactivity data (% inhibition) from tested fungal extracts to form the final input data for biochemometrics analysis. Internally cross-validated partial least squares (PLS) models were produced for each fungus using 100 iterations and a significance level of 0.05 with Sirius v.10.0 (Kvalheim et al., 2011). Built-in statistical models were used to produce S-plots identifying ions likely to be associated with antiproliferative activity in the fungal samples for targeted analysis.

### Scaled-up fermentation and metabolite isolation

The bioactive fraction from NRRL 5071 exhibited the highest production and relative purity of target metabolites (Figure S1) and was selected for scale-up fermentation and compound purification. Large-scale fermentation was conducted by inoculating an agar plug onto malt extract agar, followed by cultivation on autoclaved oats using previously described methods (Al Subeh et al., 2020). High-performance liquid chromatography (HPLC) experiments were conducted using a Varian Prostar HPLC system with a photodiode array detector and Gemini-NX C_18_ columns (Phenomenex), and data analysis was performed using Galaxie Chromatography Workstation. Normal-phase flash chromatography was performed on a CombiFlash Rf 200 using a Silica Gold column (Teledyne ISCO) and monitored by ultra-violet (UV) and evaporative light-scattering detectors. Electronic circular dichroism (ECD) spectra were acquired on an Olis DSM 17 CD spectrophotometer.

Three solid-state cultures of NRRL 5071 were chopped and extracted (~ 330 mg) following previous methods (Vandermolen et al., 2013). The defatted organic extract was reconstituted in chloroform, absorbed onto Celite 545, and purified via flash chromatography using a hexane: chloroform: methanol gradient. Fraction 4 (~ 104 mg) was further separated into six subfractions using preparative HPLC, yielding compounds** 2** and **3** (0.4 mg) from subfraction 2 and compound **1** (3.7 mg) from subfraction 3. For additional compound yield, flash chromatography fractions from NRRL 5074, 5080, and 5071 were further purified using reversed-phase preparative HPLC. The bioactive fraction from NRRL 5074 (~ 30 mg) was separated into five subfractions yielding compound **2** (6.5 mg), compound **3** (16.2 mg), and compound **1** (1.2 mg). Bioactive fractions from NRRL 5080 (10 mg) and 5071 (7 mg), were each separated into three subfractions, yielding an additional 4.8 mg of compound **1**.

### Structure elucidation of acetylstemphones

Compound elucidation employed nuclear magnetic resonance (NMR) spectroscopy, LC-HRMS/MS, ECD, encapsulated nanodroplet crystallization (ENaCt) and X-ray crystallography. LC-HRMS/MS data were obtained using an LTQ Orbitrap XL or a Q Exactive Plus mass spectrometer connected to a Waters Acquity UPLC system. NMR data were collected in CDCl_3_ on JEOL (400 or 500 MHz) and Agilent (700 MHz) NMR spectrometers.

***19-acetylstemphone G* (1):** pale yellow solid (9.7 mg); [α]D22 =  + 10.4 (*c* 0.96, MeCN) UV (MeCN) λ_max_ (log ε) 380 (2.38) nm, 283 (2.99) nm, 241 (2.76) nm, 211 (3.16) nm, 193 (3.35) nm; ^1^H (CDCl_3_, 700 MHz) and ^13^C NMR (CDCl_3_, 175 MHz), Table 1 and Figure S2; HRESIMS *m/z* 606.3286 [M + NH_4_]^+^ (calc for C_32_H_48_NO_10_, *m/z* 606.3278). ECD (MeCN) nm (Δ_ε_): 210 (+ 17.0), 277 (-4.1). 386 (+ 0.3). COSY, HSQC, HMBC, and NOESY data are provided in Figures S3–S7.

**Table 1:** ^1^H and ^13^C NMR data for compound **1** (CDCl_3_, 700 and 175 MHz, Respectively)

19-acetylstemphone G (**1**)			
Position	*δ*_C_	Type	*δ*_H_ (*J* in Hz)
1	13.3	CH_3_	1.61, buried
2	125.5	CH	5.61, q (6.5)
3	132.2	C	–
4	81.9	CH	5.43, d (9.6)
5	37.2	CH	3.35, m
6	140.4	C	–
7	135.5	C	–
7-OH	–	–	5.08, s
8	143.8	C	–
9	106.6	C	–
10	153.7	C	–
10-OH	–	–	10.69, s
11	108.0	CH	6.32, s
12	197.9	C	–
13	53.4	CH	3.19, s
14	83.7	C	–
15	37.2	CH_2_	2.05, m
16	24.7	CH_2_	1.65, 1.87, m
17	76.4	CH	3.69, dd (12.1, 4.0)
18	38.6	C	–
19	73.1	CH	5.74, t (2.7)
20	25.9	CH_2_	1.71, 1.98, m
21	80.0	C	3.46, dd (12.5, 2.8)
22	71.7	C	–
22-OH	–	–	2.40, br s
23	23.5	CH_3_	1.14, s
24	26.1	CH_3_	1.17, s
25	12.7	CH_3_	1.16, s
26	22.1	CH_3_	1.46, s
27	16.4	CH_3_	1.10, d (7.0)
28	11.5	CH_3_	1.62, s
29	169.9	C	–
30	21.3	CH_3_	1.82, s
31	169.7	C	–
32	21.0	CH_3_	2.03, s

***19-acetylstemphone B* (2):** yellow solid (6.9 mg); ^1^H NMR (CDCl_3_, 400 MHz), Figure S8; HRESMIS *m/z* 589.3018 [M + H]^+^ (calc for C_32_H_45_O_10_, *m/z* 589.3013).

***19-acetylstemphone E* (3):** yellow solid (16.6 mg); ^1^H NMR (CDCl_3_, 400 MHz), Figure S9; HRESMIS *m/z* 608.3442 [M + NH_4_]^+^ (calc for C_32_H_50_NO_10_, *m/z* 608.3435).

### Encapsulated nanodroplet crystallization (ENaCt) and crystallographic details for 19-acetylstemphone G

The crystallization of 19-acetylstemphone G (**1**) was carried out using ENaCt protocols (Tyler et al., 2020) using 12 different solvents (Table S2). The stock solutions of **1** (50 nL) were dispensed into 96-well glass plates containing either an appropriate crystallization oil (200 nL) or no oil (Table S3, Figure S10) and sealed with a glass cover slip. After 14 days, plates were assessed visually and by cross-polarized light microscopy for crystal growth. From 288 individual ENaCt experiments, 43 wells (15%) contained small single crystals suitable for X-ray diffraction analysis. A crystal of **1** grown from DMF (50 nL, *ca*. 28 mg/mL) encased in a droplet of mineral oil (Plate 1, D11) was analyzed by single crystal X-ray diffraction (Figure S11). Diffraction data were collected on a Rigaku XtaLAB Synergy diffractometer, and the structure of **1** was solved using SHELXT (Sheldrick, 2015) and refined with SHELXL (Sheldrick, 2008) through the Olex2 interface (Dolomanov et al., 2009) (Figure S12). Details regarding the data collection, solution, and refinement of **1** can be found in CIF format with the Cambridge Crystallographic Data Centre under CCDC 2303613. Crystallographic information for compound **1** is provided in Table S4.

### Cytotoxicity assays for confirmation of bioactivity of isolated compounds

Compounds** 1** and** 2** were tested against human melanoma (MDA-MB435) and human ovarian cancer cells (OVCAR3), both grown in RMPI 1640 (Thermo Fisher, #11875085) supplemented with 10% FBS (GeminiBio, #100-106) and 1% penicillin/streptomycin (P/S) (Thermo Fisher, #15070063). Compound **3** could not be tested due to its rapid interconversion to other analogs. Cells were seeded in 96-well, clear, flat-bottomed plates at 5000 cells per well and allowed to attach overnight. Compounds, dissolved in DMSO, were added to the cells for 72 h. The final DMSO concentration was 0.1%. Cellular protein content was measured using Cell Titer-Blue (Promega, #G8082) reagent for 3 h with 18 μL of reagent per well, as a measure of survival. Treatment measurements were normalized to vehicle. The analysis included three biological replicates with three technical replicates each, covering concentrations from 8 nM to 25 μM. Taxol was used as positive control (10 nM).

### Confirmation of endogenous metabolites using microextraction-enabled droplet probe mass spectrometry

Droplet probe studies were conducted with Petri dishes of the three *Aspergillus* strains used in this study. Mycelia were removed, and the agar surrounding them was analyzed (Figures S13–15) using a converted CTC/LEAP HTC PAL autosampler (LEAP Technologies, Inc.) (Cank et al., 2021; Sica et al., 2015). Briefly, microextractions were performed with 50:50 methanol: water, and the process repeated in triplicate per location before injection into the UPLC-MS. Standards were produced by depositing isolated compounds onto glass microscope slides and performing a microextraction to obtain compound retention times (Figure S16). All three compounds were observed in the agar, confirming their endogenous production.

## Results

### Metabologenomics analysis reveals hundreds of high-confidence hits

To link fungal secondary metabolites to their biosynthetic pathways, we analyzed the genomic content and metabolite profiles of 110 Ascomycetes from both public and private strain collections. Each strain underwent genome sequencing using Illumina technology, and BGCs were categorized into gene cluster families (GCFs) as previously described (Caesar et al., 2023). In this initial report, 7020 natural product BGCs were predicted across ten biosynthetic categories (NRPS, DMAT, PKS-like, HRPKS, NRPKS, PRPKS, terpene, NRPS-like, RiPP, and hybrid NRPS-PKS). Secondary metabolites were extracted from each strain grown under three different conditions and evaluated by quantitative LC–MS/MS, detecting 9301 individual ions from our 110-strain dataset. Correlations between GCF and metabolite datasets were conducted using three correlation-based integration strategies: pattern-matching, correlation scoring, and intensity ratio analysis, identifying 238 high-confidence metabolite-GCF associations. To prioritize from among these high-confidence associations, we incorporated bioactivity screening into our pipeline to focus targeted discovery efforts on bioactive metabolites.

### Bioactivity screening analysis

Many fungal natural products have demonstrated promising anticancer activity in human cancer cell lines and in mouse models with potential to enter into human clinical trials (Evidente et al., 2014). We utilized a high-throughput antiproliferative screening assay (Ayon, 2023; Ayon & Gutheil, 2019) against A549 (lung), HCT-116 (colon), LN-229 (brain/glioblastoma), and MCF7 (breast; XX genotype) cancer cell lines, and a normal cell line MCF-10A (breast; XX genotype) to identify potential anti-cancer agents using a high concentration of fungal extract (50 µg/mL) to reduce the possibility of overlooking bioactive molecules present in the extract at low abundance (Plate layout is provided in Online Resource 1, and heat maps of antiproliferative activity in Figure S17). We shortlisted the top 35 fungal extracts that showed the highest antiproliferative activity.

To further shortlist the bioactive strains, we performed a dose-dependent assay against MCF7 breast cancer cells in triplicate using doxorubicin as a positive control. From these data, we identified a subset of three bioactive strains that had similar metabolite profiles for follow up testing: *Aspergillus diversus* NRRL 5074, *A. biplanus* NRRL 5071, and *A. conjunctus* NRRL 5080. The IC_50_ of extracts against MCF7 cells for NRRL 5080, 5074, and 5071 were 14, 15, and 17 µg/mL, respectively (Figure S18).

To prioritize purification efforts towards metabolites most likely to be contributing to antiproliferative activity, the three strains were fractionated using flash chromatography, and the resulting fractions were again evaluated for antiproliferative activity at two concentrations (50 µg/mL and 16.5 µg/mL) against MCF7 cells using the MTT assay. Doxorubicin was used as positive control. From the bioactivity screening of the collected fractions, it was observed that most of the fungal extracts had one fraction that demonstrated the most potent bioactivity (~ 90% antiproliferative activity) indicating the localization of the bioactive molecule(s) in that fraction (Figure S19).

### Biochemometrics analysis

**Fig. 2:**
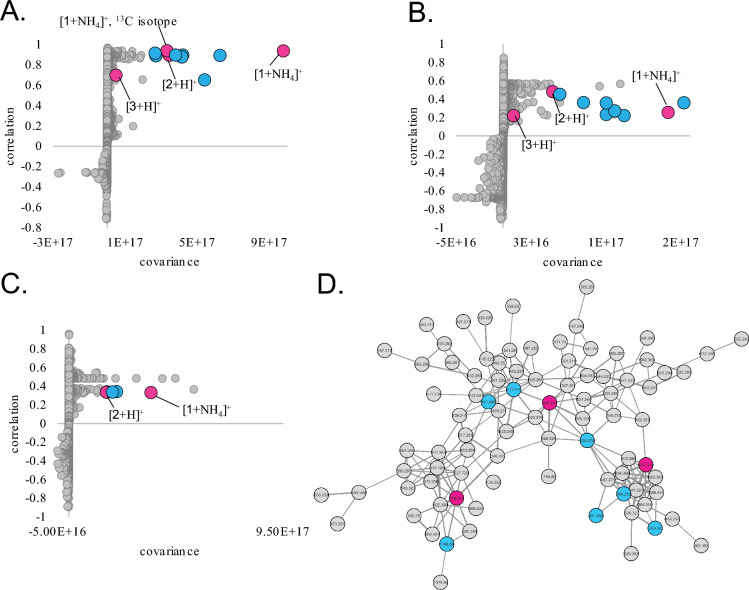
S-plots from PLS models of antiproliferative activity of **A** NRRL 5080 extract and fractions (2 component model, 93.99% independent, 99.42% dependent variation explained). **B** NRRL 5071 (2 component model, 92.20% independent, 95.51% dependent), and **C** NRRL 5074 (5 component model, 94.64% independent, 98.88% dependent). The upper right quadrants show the ions with the highest correlation to bioactivity. Compounds **1** and **2** were identified in all three S-plots among the top 10 contributors to biological activity. Points highlighted in blue were not purified, but clustered in the same molecular family as purified compounds. **D** Molecular family of 19-acetylstemphones detected by networking related MS^2^ spectra. Nodes colored in pink correspond to compounds **1–3**. Nodes highlighted in blue were not purified, but were identified through S-plot analysis as contributing to the observed antiproliferative activity and may represent additional bioactive stemphone analogs

Chemometric profiling was conducted on extracts from each of the three bioactive fungi (NRRL 5080, NRRL 5071, and NRRL 5074) as well as their fractions. Untargeted metabolomics analyses of these fractions using LC–MS yielded 7549, 5852, and 6499 total marker ions (unique retention time-*m/z* pairs) for NRRL 5080, NRRL 5071, and NRRL 5074, respectively. Individual biochemometrics analyses were completed for each strain with antiproliferative data collected at two concentrations (50 µg/mL and 16.5 µg/mL) by pairing bioactivity screening results (Figure S18) with high-resolution mass spectral data using internally cross-validated PLS analysis. The resulting biochemometrics matrices identified differences between the fungal fractions based on their antiproliferative activity. To tentatively identify the bioactive molecules responsible for this activity, S-plots (Fig. 2A–C) were generated that display the covariance and correlation of metabolite peak areas to bioactivity data. Ions located in the upper righthand quadrant of the S-plot have both high correlation and covariance and contribute most to the differentiation of bioactive and inactive fractions. Notably, S-plot analyses of the three bioactive fungi identified similar marker ions, with compound **1** ([M + NH_4_]^+^) identified among the top 4 bioactive compounds in all three fungi. In each S-plot, additional marker ions were identified as putatively bioactive that clustered together in the same molecular family using classical molecular networking analysis (Fig. 2D), suggesting that these metabolites are related and share a biosynthetic pathway. Notably, none of the target metabolites were identified using our in-house dereplication libraries nor through the GNPS platform, emphasizing their potential novelty. These marker ions were targeted for purification, structure elucidation, and downstream biosynthetic analyses.

### Identification of marker compounds from bioactive *Aspergillus* spp.

**Fig. 3:**
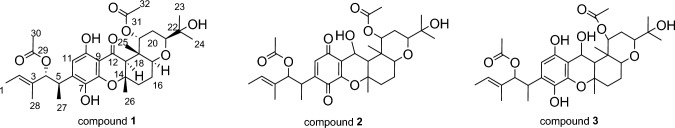
Structures of 19-acetylstemphone G (**1**), 19-acetylstemphone B (**2**), and 19-acetylstemphone E (**3**)

Additional purification of target analytes from NRRL 5080, NRRL 5071, and NRRL 5074 was completed to evaluate the accuracy of our biochemometrics predictions. Compound **1** was isolated as a pale-yellow solid. Positive ion HRMS analysis enabled assignment of the molecular formula as C_32_H_44_O_10_ [M + NH_4_]^+^ at *m/z* 606.3286 (calc. *m/z* 606.3278). Analysis of NMR data (Table 1 and Figures S2–S6) revealed that compound **1** contained three carbonyls (two esters and an α, β-unsaturated ketone), eight other sp^2^ hybridized carbons, four oxymethines, two oxygenated quaternary carbons, three aliphatic methylenes, two aliphatic methines, nine methyl groups, one aliphatic quaternary carbon and three hydroxys (Fig. 3).

A search of the Dictionary of Natural Products (2021) indicated that these data were nearly identical to those for the meroterpenoid stemphone G (Koyama et al., 2005; Yamazaki et al., 2008), but with an additional acetyl group present in compound **1**. This was supported by HMBC correlations between H-19 and the ester carbon C-31 (*δ*_*c*_169.7) and between H_3_-32 and C-31. The skeleton of the rest of the compound was confirmed by HMBC. Additional structure elucidation data are presented in the SI (Figure S7).

The geometry of the olefin between C-2 and C-3 was shown to be *E* by a NOESY correlation between H-2 and H-4 (Figure S6). NOESY correlations between H-4 and H_3_-27 set them on the same face. Cross peaks between H-19 and H_3_-23 established that they were on the same side of the molecule. Correlations from H-13 to H-17 and H-17 to H-21 established that these protons were on the same side of the molecule and opposite from H-19 and H_3_-23. Unfortunately, NOESY correlations were not observed for H_3_-25, and as such, that methyl could not be used to set the relative configuration at C-14. Using a recently described protocol (Encapsulated Nanodroplet Crystallization of organic-soluble small molecules; ENaCt) (Metherall et al., 2023; Tyler et al., 2020), which has recently found application in the crystallization of several natural products (Al Subeh et al., 2022; Straker et al., 2023), nanoscale high-throughput crystallization experiments were conducted on ~ 4.0 mg of compound **1**, resulting in crystalline material. Suitable single crystals were selected for X-ray diffraction analysis, showing that compound **1** had crystallized as a solvate with two molecules of DMF. Compound **1** crystallized in the monoclinic space group *P*2_1_ with two formula units per unit cell and one per asymmetric unit. While the absolute configuration could not be determined based on anomalous scattering (Flack parameter 0.3(3)), the X-ray structure did confirm the molecular connectivity and allowed assignment of the relative configuration, allowing the relative stereochemistry of position C-14 to be determined (Figure S12). This also confirmed that the relative configuration of compound **1** was identical to the reported configuration of stemphone G, by comparison with the known crystal structure of stemphone (Huber et al., 1974; Yamazaki et al., 2008). ECD data were collected, and the predicted spectra were calculated based on the two possible absolute configurations. The measured ECD spectrum matched the calculated one, where the configurations were 4*S*, 5*S*, 13*R*, 14*R*, 17*R*, 18*R*, 19*S*, 21*R* (Figure S20); compound **1** was ascribed the name 19-acetylstemphone G. Although this compound has been previously synthesized (Aurora Fine Chemicals, Product Number 185.827.908), this represents its first discovery from a natural source.

Two other major peaks were observed during preparative HPLC separation, which upon MS analysis revealed their molecular formulas and *m/z* values to be C_32_H_45_O_10_ as [M + H]^+^ (calc. *m/z* 589.3013) and C_32_H_50_NO_10_ as [M + NH_4_]^+^ (calc. *m/z* 608.3435). Comparison of the ^1^H NMR spectra for compounds **2** and **3** (Figures S8–S9) to published data confirmed (**2**) and (**3**) as 19-acetylstemphones B and E, respectively (Fig. 3). These three stemphone analogs were observed in the extracts of all three fungal strains used in this study (Figure S1). Interestingly, conversion of these analogs was observed when the extracts were left in methanol for a period of 2 days, as observed by the presence of a peak for only compound **1** during HPLC analysis, which indicates conversion of 19-acetylstemphone E (**3**) to 19-acetylstemphone B (**2**), and finally to 19-acetylstemphone G (**1**). This indicates that compound **1** (19-acetylstemphone G) is the most stable of the three acetylstemphone analogues.

To ascertain the presence of acetyl moiety on the stemphone analogs as endogenous fungal metabolites and not an artefact of the extraction and purification process (Capon, 2020) and also to confirm the presence of all three acetylstemphones in fungi as a proof of their interconversion, droplet probe mass spectrometry analysis (Oberlies et al., 2019; Sica et al., 2015) was conducted. This technique makes a microextraction of the fungal culture, directly from the petri dish, and then analyzes it by LC–MS. Acetylstemphones G (**1**), B (**2**) and E (**3**) were all observed during the analysis, indicating that they are endogenous fungal metabolites (Figures S13–S16).

### Confirmation of bioactivity of the isolated stemphone analogs

The antiproliferative activities of compounds **1** and **2** were tested against human melanoma cancer cells (MDA-MB-435) and human ovarian cancer cells (OVCAR3) using previously described protocols (Gurgul et al., 2024). **1** and **2** were approximately equipotent, with IC_50_ values of 4.5 ± 0.1 µM and 3.6 ± 0.2 µM against OVCAR3 and IC_50_ values of 4.4 ± 0.1 µM and 4.20 ± 0.02 µM against MDA-MB-435 for compounds **1** and **2**, respectively (Table S5).

### Binary correlation to identify stemphone biosynthetic pathway

To identify the stemphone gene cluster family (GCF) the pattern-matching and correlation scores for each ion were analyzed (Table S6, Figure S21). GCFs are named by the type of biosynthetic pathway followed by an underscore and the in-house GCF identifier described in our previous publication (Caesar et al., 2023). Eighteen PKS-containing GCFs had high correlation scores (> 100) to the 19-acetylstemphones, likely because the three producing strains share many BGCs and metabolite products. The stemphone GCF was identified by examining predicted GCFs for 19-acetylstemphones G, B, and E (compounds **1–3**). Analysis of the three stemphone-producing strains revealed four PKS-containing GCFs that were common to all three producing strains (PRPKS_244, HRPKS_363, NRPKS_110, and NRPKS_208; Table 2).

**Table 2:**
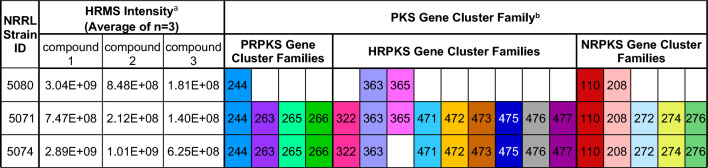
*Aspergillus* strains that produce the 19-acetylstemphones (compounds **1–3**) and their potential PKS-containing biosynthetic gene clusters

^a^HRMS = high resolution mass spectrometry. Compound **1 = **19-acetylstemphone G, compound **2** = 19-acetylstemphone B, compound **3** = 19-acetylstemphone E

^b^Gene cluster families are named by BiosyntheticType_IdentifyingNumber (*e.g.,* PRPKS_244). The numbers indicated here refer to the identifying number for PKS-containing GCFs. All GCFs were grouped as part of a previous project (Caesar et al., 2023)

Structurally, stemphones are related to the meroterpenoids arthripenoids A-F (Figure S22), compounds previously isolated from *Arthrinium* sp. NF2194 and which have a published biosynthetic pathway (Zhang et al., 2018). The meroterpenoid scaffold of arthripenoids A-F results from the biosynthesis of 2,4,-dihydroxy-5-alkylbenzoic acid, which undergoes oxidative decarboxylation, farnesyl transfer, and terpene cyclization. Because compounds **1**–**3** share these structural features with the arthripenoids, we expected the BGC responsible for their production to encode highly similar enzymes. Comparison of our top-ranked GCF candidates to the arthripenoid pathway revealed PRPKS_244 as the best candidate GCF for the stemphones, with all BGCs belonging to the PRPKS_244 GCF including partially-reducing polyketide synthases (PR-PKS), non-reducing polyketide synthases (NR-PKS), polyprenyl transferases, and a terpene cyclases that were found to have moderate sequence similarity to their arthripenoid counterparts (Table S7, Figure S23). Accessory enzymes, including two monooxygenases, a P450, and an acetyltransferase were also identified in the stemphone BGC with a high degree of similarity to those found in the arthripenoid cluster (Tables S7–S8).

**Fig. 4:**
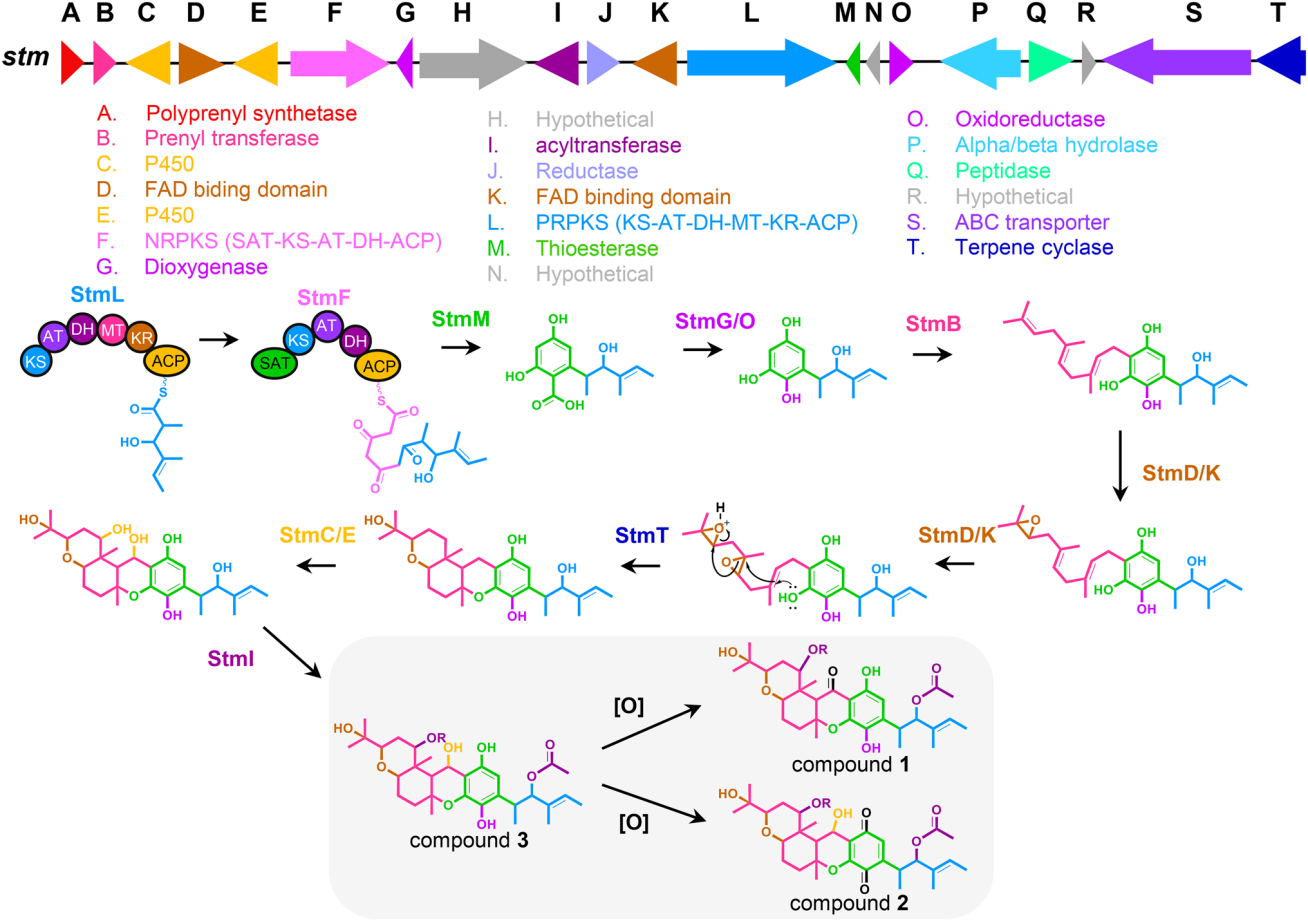
Proposed biosynthesis of 19-acetylstemphones. In final structures, R=H for stemphones and R=COCH_3_ for acetylstemphones

Based on our data and the published arthripenoid pathway, we propose the biosynthetic scheme in Fig. 4. We suggest stemphone biosynthesis begins with the PR-PKS StmL, which catalyzes the biosynthesis of a partially reduced triketide which is further extended by the NR-PKS StmF. Unlike its arthripenoid NR-PKS counterpart AtnG, StmF does not have a thioesterase domain. We hypothesized instead that StmM acts as a freestanding thioesterase allowing the product to be released from the PKS enzymes. The polyketide product then undergoes an oxidative decarboxylation step catalyzed by oxidoreductase StmO and/or StmG. Following decarboxylation, StmA provides a farnesyl group to the polyprenyl transferase StmB to be incorporated at the C-9 position. Prior to cyclization the product undergoes two successive epoxidation reactions carried out by FAD-dependent monooxygenases StmD and StmK. The tricyclic ring formation is then initiated by StmT, a terpene cyclase, following which the cytochrome P450s StmC and/or StmE hydroxylate C-12 and C-19. Finally, the acetyltransferase StmI acetylates oxygens at the C-4 and C-19 positions.

## Discussion

Metabologenomics workflows often yield hundreds or thousands of correlated metabolite-BGC pairs (Navarro-Muñoz et al., 2020; Ren et al., 2020; van der Hooft et al., 2020), and prioritizing these for follow up studies can be a daunting task. Several approaches to ameliorate this challenge have been explored, including those based on dereplication, microbial taxonomy, coevolutionary principles, molecular networking, chemical class matching, and/or paired genome-metabolite databases, and new computational tools to improve efficiency are under continual development (Avalon et al., 2022; Louwen et al., 2023). This work serves as a proof-of-concept for the viability of using bioactivity-driven metabologenomics for prioritizing metabolites for targeted analysis. Through this work, we identified three novel stemphone analogs, 19-acetylstemphones G (**1**), B (**2**) and E (**3**), confirmed their antiproliferative activity against human melanoma and ovarian cancer cell lines, and delineated a likely biosynthetic pathway for their production.

In order for paired -Omics strategies to identify a metabolite’s biosynthetic pathway, the genes involved in biosynthesis must be expressed (Kjærbølling et al., 2019). It is well documented that many BGCs in fungi are silent under standard laboratory conditions (Gilchrist et al., 2018) and that manipulation of culture conditions can activate fungal BGCs (Mózsik et al., 2022). Indeed, during our scale up effort to isolate compounds **1–3**, we witnessed a pronounced effect of growth condition on metabolite production. When media were prepared with nano-pure water, the fungi yielded an increased amount of stemphone analogs compared to when the media was prepared with deionized water (Figure S24). The appearance and the color of the fungal culture and the extracts were different to the naked eye as well. This difference in the metabolite profile emphasizes the impact of even seemingly minor changes to media conditions on the expression of BGCs encoding bioactive metabolites.

Solvents used during extraction and purification of metabolites play an important role in natural product chemistry, often leading to the formation of “artifacts” through chemical reactions of naturally occurring metabolites and the solvent (Maltese et al., 2009). The presence of acetyl group on the purified stemphone analogs was confounding, and it was important to determine if it was part of the endogenous fungal secondary metabolite profile or caused during the extraction or purification process, particularly due to our observation that compound **3** converted to **2** and then to **1** in methanol. Addition of acetyl groups (i.e., esterification of a hydroxy group) can occur in the presence of acetic acid (Anbu et al., 2019) or by external stimuli including changes in pH, temperature, or exposure to light, air, common organic solvents, and even chromatography media (Capon, 2020). Conversely, acetyltransferase enzymes are present in many fungi that can also acetylate fungal metabolites (Sharma et al., 2020), including the acetyltransferase StmI in our proposed biosynthetic pathway (Fig. 4). In order to determine if the acetylation of compounds **1**–**3** resulted from our extraction/purification procedures or was naturally occurring, we used droplet probe LC–MS (Oberlies et al., 2019), an in situ analytical technique that allows direct analysis of constituents from the sample without requiring lengthy extraction procedures that may introduce artifacts. This analysis revealed the presence of all three stemphone analogs in the sample, confirming their identity as endogenous fungal secondary metabolites (Figures S13–15).

While most fungal GCFs contain BGCs from fungi belonging to a single species or genus, BGCs encoding stemphones and the highly related arthripenoids and nectripenoids (Table S7) have a remarkably extensive phylogenetic distribution, with highly similar metabolites and BGCs identified in two Leotiomyceta classes: Eurotiomycetes and Sordariomycetes (Zhang et al., 2018). Moreover, the first stemphone was isolated from a *Stemphylium* sp. (class Dothidiomycetes), and Zhang et al. have also identified highly related putative meroterpenoid BGCs in three *Bipolaris* spp. (class Dothidiomycetes) (Zhang et al., 2018). More recently, Han and coworkers (Han et al., 2020) have isolated both arthripenoids and cochlioquinones from *Bipolaris sorokiniana* and have identified several similar biosynthetic pathways across the *Bipolaris* genus. These results suggest that BGCs encoding bioactive stemphones and related metabolites span at least three fungal classes. The presence of highly similar biosynthetic pathways across such broad phylogenetic distances is extremely rare (~ 0.75% of total fungal GCFs) (Robey et al., 2021) and suggests that meroterpenoid BGCs in taxonomically distant fungi may be acquired through horizontal gene transfer or convergent evolution (Zhang et al., 2018). Other metabolites that have been purified from multiple taxonomic classes encode the bioactive equisetins, PR-toxins, cytochalasins, and chaetoglobosins, and the associated biosynthetic pathways contain significant variation in tailoring enzyme composition resulting in scaffold diversification (Robey et al., 2021). The discovery of the stemphone biosynthetic pathway provides growing evidence for the biosynthetic diversity represented within “multi-class” GCFs, and emphasizes the promise of exploring cross-class GCF-metabolite pairs to discover new analogs of bioactive metabolites.

## Conclusion

This study highlights the potential of integrated -Omics approaches for identifying biologically active fungal secondary metabolites and their biosynthetic pathways. We demonstrated the effectiveness of a bioactivity-driven metabologenomics workflow for prioritizing bioactive metabolite-BGC pairs for targeted study, leading to the discovery of three new antiproliferative stemphone analogs. Using our metabologenomics database, we identified the BGC likely for their production and proposed a biosynthetic pathway for these compounds, shedding light on the biosynthesis of this intriguing class of natural products whose distribution across vast taxonomic distances raises questions about the evolution of these metabolites in the fungal kingdom. These findings also underscore the importance of growth conditions and extraction procedures in natural products studies, as these factors can significantly impact metabolite production and chemical modifications. Overall, this work serves as a proof-of-concept for the bioactivity-driven metabologenomics workflow as a valuable framework for the targeted discovery of bioactive compounds and their biosynthetic machinery from the largely unexplored world of fungal secondary metabolism.

## Supplementary Information

Below is the link to the electronic supplementary material.

Supplementary file1 (DOCX 70941 KB)

Supplementary file2 (XLSX 15 KB)

## Data Availability

All genomes that were sequenced for this work (as part of our previous publication) are available via NCBI under BioProject PRJNA852164. The metabolomics data (as.mzXML files) for the 110-strain dataset are available via the MassIVE repository under accession no. MSV000089848 and that for the 3 strains, the primary focus of this article, are available via the MassIVE repository under Accession No. MSV000094411. NMR data are available as an NP-MRD deposition under the ID numbers (NP0332825, NP0332827, and NP0332826 for compounds **1**-**3**, respectively). The supplementary crystallographic data for this paper are provided under Cambridge Crystallographic Data Centre under CCDC 2303613. These data are provided free of charge by the joint Cambridge Crystallographic Data Centre and Fachinformationszentrum Karlsruhe Access Structures service. Additional data can be made available upon request.
