# Supplementary material for: Bioactivity-driven fungal metabologenomics identifies antiproliferative stemphone analogs and their biosynthetic gene cluster
Source: Metabolomics. 2024 Aug 2;20(5):90. doi: 10.1007/s11306-024-02153-8 (PMC11296971; doi:10.1007/s11306-024-02153-8)
Supplement: Supplementary file 1 — Supplementary file1 (DOCX 70941 KB) [file 11306_2024_2153_MOESM1_ESM.docx]

**Supplementary Information**

**for**

**Bioactivity-driven metabologenomics identifies antiproliferative stemphone analogs and their biosynthetic gene cluster**

Navid J. Ayon^1,2^, Cody E. Earp^3^, Raveena Gupta^1^, Fatma A. Butun^1,2^, Ashley E. Clements^4^, Alexa G. Lee^4^, David Dainko^1^, Matthew T. Robey^5^, Manead Khin^6^, Lina Mardiana^7,8,9^, Alexandra Longcake^7^, Manuel E. Rangel Grimaldo^3^, Michael J. Hall^7^, Michael R. Probert^7^, Joanna E. Burdette^6^, Nancy P. Keller^10,11^, Huzefa A. Raja^3^, Nicholas H. Oberlies^3^, Neil L. Kelleher^1,2,5^, Lindsay K. Caesar^4*18^

^1^Department of Chemistry, Northwestern University, Evanston, Illinois, USA

^2^Proteomics Center of Excellence, Northwestern University, Evanston, Illinois, USA

^3^Department of Chemistry and Biochemistry, University of North Carolina at Greensboro, Greensboro, North Carolina, USA

^4^Department of Chemistry and Biochemistry, James Madison University, Harrisonburg, Virginia, USA

^5^Department of Molecular Biosciences, Northwestern University, Evanston, Illinois, USA

^6^College of Pharmacy – Pharmaceutical Science, University of Illinois Chicago, Chicago, Illinois, USA

^7^Chemistry, School of Natural and Environmental Sciences, Newcastle University, Newcastle upon Tyne NE1 7RU, UK

^8^Department of Chemistry, Universitas Indonesia, Depok, Jawa Barat, Indonesia

^9^Indicatrix Crystallography, Newcastle University, Newcastle upon Tyne NE1 7RU, UK

^10^Department of Medical Microbiology and Immunology, University of Wisconsin-Madison, Madison, Wisconsin, USA

^11^Department of Bacteriology, University of Wisconsin-Madison, Madison, Wisconsin, USA

^*^Corresponding author: caesarlk@jmu.edu (L.K.C.)

**Table of Contents**

[**Supplementary Materials and Methods** 5](#_Toc169635445)

[**Table S1.** MZMine Processing Parameters. 10](#_Toc169635446)

[**Figure S1.** UPLC chromatograms of flash chromatography fractions from NRRL 5071, 5074, and 5080. 11](#_Toc169635447)

[**Figure S2**. 1H and 13C NMR spectra of 19-aceytlstemphone G (compound **1**; 700 MHz, CDCl_3_). 12](#_Toc169635448)

[**Figure S3.** COSY NMR spectrum of 19-acetylstemphone G (compound **1**; 700 MHz, CDCl_3_). 13](#_Toc169635449)

[**Figure S4**. HSQC NMR spectrum of 19-acetylstemphone G (compound **1**; 700 MHz, CDCl_3_). 14](#_Toc169635450)

[**Figure S5**. HMBC NMR spectrum of 19-acetylstemphone G (compound **1**; 700 MHz, CDCl_3_). 15](#_Toc169635451)

[**Figure S6.** NOESY NMR spectrum of 19-acetylstemphone G (compound **1**; 700 MHz, CDCl_3_). 16](#_Toc169635452)

[**Figure S7.** Key HMBC correlations for 19-acetylstemphone G (compound **1**). 17](#_Toc169635453)

[**Figure S8.** 1H NMR spectrum of 19-acetylstemphone B (compound **2**; 400 MHz, CDCl_3_). 18](#_Toc169635454)

[**Figure S9.** 1H NMR spectrum of 19-acetylstemphone E (compound **3**; 400 MHz, CDCl_3_). 19](#_Toc169635455)

[**Table S2.** Preparation of stock solutions of 19-acetylstemphone G (compound **1**) for ENaCt experiments. 20](#_Toc169635456)

[**Table S3.** Encapsulation oils for ENaCt Experiments. 21](#_Toc169635457)

[**Figure S10.** Plate setup and crystallization results for the ENaCt protocol used, detailing the type, amount and concentration of the respective stock solution and oil as well as the classification of the wells after two weeks. 22](#_Toc169635458)

[**Figure S11.** An image of Plate 1 well D11, with a scale bar. 24](#_Toc169635459)

[**Figure S12.** X-ray crystallographic structure 19-acetylstemphone G (compound **1**), showing DMF molecules within the structure (top) and overlaid with key NOESY correlations (bottom). 25](#_Toc169635460)

[**Table S4.** Crystallographic parameters for compound **1**. 26](#_Toc169635461)

[**Figure S13.** UPLC chromatograms from droplet probe analysis of NRRL 5071. 27](#_Toc169635462)

[**Figure S14.** UPLC chromatograms from droplet probe analysis of NRRL 5074. 28](#_Toc169635463)

[**Figure S15.** UPLC chromatograms from droplet probe analysis of NRRL 5080. 29](#_Toc169635464)

[**Figure S16.** UPLC chromatograms from droplet probe analysis of standards of compounds **1**, **2**, and **3**. 30](#_Toc169635465)

[**Figure S17.** Heatmaps of secondary high-throughput screening of fungal strains against (A) MCF10a human mammary epithelial, (B) A549 lung cancer, (C) HCT 116 colon cancer, (D) LN229 glioblastoma, and (E) MCF7 breast cancer cell lines. 31](#_Toc169635466)

[**Figure S18.** Dose response curves of (A) NRRL 5071, (B) NRRL 5074, and (C) NRRL 5080 against MCF-7 cells. 32](#_Toc169635467)

[**Figure S19.** Bioactivity of flash chromatography fractions from (A) NRRL 5071, (B) NRRL 5074, and (C) NRRL 5080 against MCF-7 cells used for biochemometrics analysis. 33](#_Toc169635468)

[**Figure S20**. Comparison of the experimental and calculated ECD spectra for 4S, 5S, 13R, 14R, 17R, 18R, 19S, 21R 19-acetylstemphone G (compound **1**). 34](#_Toc169635469)

[**Table S5.** Antiproliferative/cytotoxicity data for compounds **1** and **2** against OVCAR3 and MDA-MB-435 cell lines. 35](#_Toc169635470)

[**Table S6:** Correlation scores for the 19-acetylstemphones with biosynthetic gene cluster families. 36](#_Toc169635471)

[**Figure S21.** Compiled metabolite-GCF correlations for acetylstemphones. 37](#_Toc169635472)

[**Figure S22.** Structurally-related meroterpenoids sharing a 6/6/6/6-tetracyclic ring system consisting of sesquiterpenoid and polyketide components 38](#_Toc169635473)

[**Table S7.** Comparative analysis of stm gene cluster and atn gene cluster. 39](#_Toc169635474)

[**Figure S23.** Schematic representation of the members of the PRPKS_244 gene cluster family and the amino acid sequence identities. 40](#_Toc169635475)

[**Table S8.** Annotation of the *stm* gene cluster from *Aspergillus biplanus* NRRL 5071. 41](#_Toc169635476)

[**Figure S24.** Effect of growth conditions on metabolomic profiles. 42](#_Toc169635477)

# **Supplementary Materials and Methods**

**Fungal growth and metabolome extraction:** As previously described (Caesar et al., 2023), fungal strains were collected from public and private sources including the Agricultural Research Service Culture Collection (NRRL), the American Type Culture Collection (ATCC), and the Central Bureau of Fungal Cultures (CBS) collections for metabologenomics analysis. Fungi were grown in three different media, namely rice, old-fashioned Quaker oats and Cheerios^TM^ (original) to maximize secondary metabolite biosynthesis (Graf et al., 2020). First, glycerol stocks or agar plugs of each fungus were inoculated onto potato dextrose agar (PDA) plates and incubated at 21 °C. When mycelial mats were fully grown at 5-7 days, agar plugs were cut and transferred to 10 mL of YESD broth (2% soy peptone [20 g], 2% dextrose [20 g], and 1% yeast extract [10 g]) and cultivated at 21 °C at 150 rpm for 3 days to prepare seed cultures. Three seed cultures were prepared for each fungus and used to inoculate three 250 mL Erlenmeyer flasks containing autoclaved rice (15 mL deionized water per 10 g rice), oats (20 mL deionized water per 10 g oats), or Cheerios (alone, no water) which were inoculated at 21 °C for 2-5 weeks (Al Subeh et al., 2021).

Secondary metabolite extraction was performed according to established methods (Al Subeh et al., 2021; Graf et al., 2020). Briefly, a 1:1 mixture of methanol and chloroform (60 mL) was added to each flask and the culture was chopped with a spatula, followed by brief sonication. The flasks were left overnight at room temperature. The next day, cultures were briefly sonicated again and filtered under vacuum. The filtrate was collected, after which 90 mL of chloroform and 150 mL of water were added and transferred to a separatory funnel, where the organic layer was collected and evaporated under N_2_. The dried extract was reconstituted with a 100 mL of a 1:1 mixture of methanol and acetonitrile to which was then added 100 mL hexanes, transferred to a separatory funnel and mixed by vigorous shaking. The defatted organic layer was then collected and dried under N_2_ and subjected to LC-MS/MS analysis and bioactivity screening.

**Acqusition and dereplication of metabolomics datasets:** Dried fungal extracts were reconstituted in methanol at a concentration of 1 mg/mL and transferred to filter vials for liquid chromatography tandem high-resolution mass spectrometry (LC-MS/MS) based untargeted metabolomics analysis as previously described (Caesar et al., 2023). All LC-MS/MS analyses were performed on a Thermo Q Exactive mass spectrometer (MS) using electrospray ionization in positive mode coupled with an Agilent 1290 Infinity II ultrahigh performance liquid chromatograph on a Kinetix C_18_ column (50 mm × 2.1 mm, 1.3 µm, 100 Å particle size). The flow rate, column oven temperature, and injection volume were 0.3 mL/min, 40 °C and 5 µL, respectively. Using a binary solvent system consisting of 5% acetonitrile in water with 0.1% formic acid (mobile phase A) and 100% acetonitrile with 0.1% formic acid (mobile phase B), the gradient increased from 5-100% B from 0 to 8.00 min, after which it was held at 100% B from 8.00-9.00 min. Starting chromatographic conditions were re-established from 9.00-9.20 minutes and held for the final 0.80 minutes. Other MS parameters were: capillary temperature 320 °C, sheath gas 10 (arbitrary unit) and spray voltage 3.6 kV. Full MS spectra were acquired at a resolution of 17,500 for the mass range of 150-2,000 *m/z* with data-dependent fragmentation experiment for MS^2^ data collection with the top five ions of each scan. A normalized collision energy of 25 was used for higher-energy collisional dissociation (HCD). Feature detection and processing of LC-MS data were performed using MZmine v. 2.53 (Pluskal et al., 2010) with parameters described in our previous publication (Caesar et al., 2023).

To dereplicate known compounds in our extracts, retention times, *m/z* values, MS^2^ fragmentation patterns, and/or UV absorbance patterns were compared to in-house dereplication libraries containing data for more than 700(?) authenticated standards. Using this data, we were able to identify over 350 known metabolites in our fungal extracts (Caesar et al., 2023; El-Elimat et al., 2013; Paguigan et al., 2017).

**Primary high throughput screening assay for identifying bioactive fungal extracts:** To striate the more than 200 significant natural product-BGC pairs identified in our previous study (Caesar et al., 2023), fungal extracts were subjected to bioactivity screening against A549 (lung; XY genotype), HCT-116 (colon; XY genotype), LN-229 (brain/glioblastoma; XX genotype), and MCF7 (breast; XX genotype) cancer cell lines, and a normal cell line MCF-10A (breast; XX genotype). Cells were grown until 80-85% confluency in cell culture flasks (Corning, TC-treated, 75 cm^2^) and 5,000 cells were transferred to each well of 384 well plates (Corning # 3985) on day 1 and incubated at 37 °C in presence of 5% CO_2_ with a water bath to maintain humidity inside the incubator. After 24 hours, extracts (prepared at 50 mg/mL in DMSO) were added to the cells for a final concentration of 50 µg/mL (and final DMSO concentration of 1%) using the Echo 550 Acoustic liquid transfer system (Labcyte Inc.). Doxorubicin was used as a positive control at concentrations of 12.5, 25, and 50 µM. Plates were then incubated for 48 hours at 37 °C after which CellTiter-Glo® 2.0 reagent was added (equal volume of the cell content) and cell proliferation was calculated through measurement of luminescence at 600 nm using a BioTek Epoch Microplate Spectrophotometer (Agilent) that represents the number of metabolically active cells by quantifying ATP as an indication of the number of viable cells in each well. Extracts that showed antiproliferative activity against at least one cancer cell line were then tested at concentrations of 25, 50 and 100 µg/mL to identify the most bioactive strains.

**Fractionation and antiproliferative activity of bioactive fungal extracts:** Of our 110 strains, 12 had potent antiproliferative activity against our cancer cell line panel. From these, three of the most potent extracts had similar chemical and sensitivity profiles for all 5 cell lines and were separated using flash chromatography. These strains were *Aspergillus biplanus* NRRL 5071, *A. diversus* NRRL 5074, and *A. conjunctus* NRRL 5080. Crude extracts (ranging from 200 µg – 4 mg) were subjected to normal-phase flash chromatography using a Teledyne ISCO CombiFlash NextGen 300 system and evaluated using ultraviolet absorbance at 254 and 280 nm using a 45-min ethyl acetate/hexane/methanol gradient on a silica 4-g gold column (Teledyne ISCO) at a flow rate of 18 mL/min. Fractions were collected and combined based on the presence of individual peaks in the chromatograms for bioactivity testing and metabolomics analysis.

To generate bioactivity data for biochemometrics correlations, flash chromatography fractions collected from the bioactive strains were tested against MCF7 cells with doxorubicin as a positive control at concentrations of 50 µg/mL and 16.5 µg/mL. Cells were grown until 80-85% confluency in cell culture flasks (Corning, TC-treated, 75 cm^2^) and 10,000 cells were transferred to each well of 96-well plates (Fisher # FB012931) on day 1 and incubated at 37 °C in presence of 5% CO_2_ with a water bath to maintain humidity inside the incubator. After 24 hours, extracts were added to the cells at a final concentration of 16.5 µg/mL and 50 µg/mL in triplicate. The plates were then incubated for a 48-hour treatment period, after which cell proliferation was measured using a 3-[4,5-dimethylthiazol-2-yl]-2,5 diphenyl tetrazolium bromide (MTT) assay. Briefly, the MTT reagent was added after 48 hours and incubated for 4 hours, after which the solubilizing agent DMSO was added. Plates were incubated overnight and read using a BioTek Epoch Microplate Spectrophotometer (Agilent) at 600 nm to determine cell viability.

**LC-HRMS/MS-based correlation of metabolomics and bioactivity datasets:** To generate metabolomics data required for biochemometrics correlations, LC-MS/MS spectra of flash chromatography fractions were acquired using the same instrument parameters described above. Files were converted to .mzXML file format using ProteoWizard (Chambers et al. 2012), following which feature detection and processing of LC-MS data were performed using MZmine v. 2.53 (Pluskal et al., 2010) using modified parameters outlined in **Table S1** to create a feature table consisting of *m/z* values, retention times, and peak areas of detected ions for biochemometrics correlations. Additionally, .mzXML files were uploaded to the Global Natural Products Social Molecular Networking (GNPS) site (Wang et al., 2016) to visualize molecular families. For data curation, all MS^2^ fragment ions within ± 17 Da of the precursor *m/z* were removed and only the top six fragment ions were kept and compared for the analysis. The precursor ion mass tolerance was set to 2.0 Da and a MS/MS fragment ion tolerance of 0.5 Da. Ions were grouped into the same molecular family that have a cosine score (similarity score) above 0.70 and more than six matched fragment peaks. Further, edges between two nodes were kept in the family if and only if each of the nodes appeared in each other’s respective top 10 most similar nodes. The maximum size of the molecular family was set to 100, which was used as a threshold to remove the lowest scoring edges from the families until their size was below this threshold.

The metabolite feature table containing *m/z* values, retention times, and peak heights from flash chromatography fractions (described in “fractionation and antiproliferative activity of bioactive fungal extracts section above) was merged with the bioactivity data (% inhibition) from fungal extracts tested at 16.7 µg/mL and 50 µg/mL to form the final input data for biochemometrics analysis. Statistical analysis was conducted using Sirius v.10.0 (Kvalheim et al., 2011). Internally cross-validated partial least squares (PLS) models were produced for each fungus using 100 iterations and a significance level of 0.05. Built-in statistical models were used to produce S-plots identifying ions likely to be associated with antiproliferative activity in the fungal samples for targeted analysis. Notably, none of the ions identified as our top targets for isolation were identified using any of our dereplication strategies, emphasizing their potential novelty.

**Scaled-up fermentation and metabolite isolation:** The target ions identified from the S-plot analysis were present in all three bioactive strains, but the bioactive fraction from *A. biplanus* NRRL 5071 had the highest production and relative purity of target metabolites (**Figure S1**) and was selected for scale-up fermentation and purification of the bioactive compounds. Fungal culture, fermentation and extraction were carried out following the details discussed in the “fungal growth and metabolome extraction” section above with slight modifications. Briefly, we began by inoculating glycerol stock or agar plugs on malt extract agar (MEA, Difco). For large-scale fermentation, 3-6 flasks of autoclaved breakfast oats (old-fashioned Quaker oats) were grown for each strain using previously described methods (Al Subeh et al., 2020). To initiate the seed cultures, an agar plug from the leading edge of the MEA culture was transferred to a sterile tube with 10 mL of YESD per 1 L of distilled or nanopure water. The YESD culture was grown for seven days on an orbital shaker (100 rpm) at room temperature (∼23 °C). To prepare solid-state grain fermentation growth, the YESD seed culture was used to inoculate 10 g of autoclaved breakfast oats (old-fashioned Quaker oats) prepared with 17 mL of distilled water in 250 mL Erlenmeyer ﬂasks. The cultures were incubated under ambient light with 12 hours light and 12 hours dark cycle.

Analytical and preparative high-performance liquid chromatography (HPLC) experiments were performed using a Varian Prostar HPLC system equipped with ProStar 210 pumps and a Prostar 335 photodiode array detector (PDA), with data collected and analyzed using Galaxie Chromatography Workstation software (version 1.9.3.2, Varian Inc.). All chromatography was conducted on Gemini-NX C_18_ analytical (50 x 4.6 mm, 5 µm) or preparative (250 x 21.2 mm, 5 µm) columns (all from Phenomenex), with acidified water (0.1% formic acid) as solvent A and acetonitrile (0.1% formic acid) as solvent B. Flash chromatography was performed on a CombiFlash Rf 200 using a Silica Gold column (from Teledyne ISCO) and monitored by ultra-violet (UV) and evaporative light-scattering detectors (ELSD). Electronic circular dichroism (ECD) spectra were acquired on an Olis DSM 17 CD spectrophotometer.

The three flasks of solid-state fermentation cultures of *A. biplanus* NRRL 5071 were chopped into small pieces using a spatula, and extracted according to previous methods, described above in the “fungal growth and metabolome extraction” section above (Vandermolen et al., 2013). The defatted organic extract (~330 mg) was reconstituted in chloroform and absorbed onto Celite 545. This sample was purified using flash chromatography with a solvent gradient of hexanes: chloroform: methanol at an 18 mL/min flow rate and 72 column volumes to yield four fractions using a Silica Gold columns (Teledyne ISCO). Fraction 4 (~104 mg) was further separated into six subfractions using preparative HPLC with a gradient solvent system that increased linearly from 50:50 to 75:25 acetonitrile: water (acidified with 0.1% formic acid) over 20 min at a flow rate of 21.2 mL/min on a C_18_ column (Phenomenex, 250 x 21.2 mm, 5 µm). Subfraction 2 yielded compounds **2** and **3** (0.4 mg), which eluted at 10 min. Subfraction 3 yielded compound **1** (3.7 mg), which eluted at 12 min.

To obtain more compound for structure elucidation, flash chromatography fractions from *A. diversus* NRRL 5074, *A. conjunctus* NRRL 5080, and *A. biplanus* NRRL 5071 described in the “Fractionation and antiproliferative activity of bioactive fungal extractions” section above were used for further purification of bioactive constituents. Extracts were separated using a preparative HPLC on a C_18_ column (Phenomenex, 250 x 21.2 mm, 5 µm) with a gradient solvent system that increased linearly from 35:65 (acetonitrile: water (acidified with 0.1% formic acid) to 65:35 over 20 min and holding at 65:35 for 15 min at a flow rate of 21.2 mL/min. The bioactive fraction from NRRL 5074 (~30 mg) was separated into five subfractions yielding compound **2** (6.5 mg, eluting at 18.9 min), compound **3** (16.2 mg, eluting at 20.5 min), and compound **1** (1.2 mg, 24.2 min). For NRRL 5080, bioactive fraction 2 (10 mg) was separated into three subfractions, yielding compound **1** (2.7 mg), which eluted at 24.6 min. Finally, the bioactive fraction from NRRL 5071 (7 mg) was separated into three subfractions. Subfraction 2 yielded compound **1** (2.1 mg), which eluted at 24.6 min.

**Structure elucidation of acetylstemphones:** Structure elucidation of the isolated compounds was conducted using nuclear magnetic resonance (NMR) spectroscopy, LC-HRMS/MS, ECD, encapsulated nanodroplet crystallization (ENaCt) and single crystal X-ray crystallography. LC-HRMS/MS data were collected on either a Thermo Fisher Scientific LTQ Orbitrap XL mass spectrometer or a Thermo Fisher Scientific Q Exactive Plus mass spectrometer, both equipped with a heated electrospray ionization (HESI) source (Thermo Fisher Scientific) and connected to a Waters Acquity ultra performance liquid chromatography (UPLC) system. A Phenomenex Kinetix C_18_ column (50 x 2.1 mm, 1.3 µm), heated to 40 °C, was operated at a flow rate of 0.3 mL/min with a gradient system of 15:85 to 100:0 of acetonitrile: water (0.1% formic acid) over 10 min. MS data were collected from 150 to 2000 m/z in the positive ion mode. NMR data were obtained using either a JEOL SS-400 MHz NMR spectrometer operating at 400 MHz for ^1^H and 100 MHz for ^13^C, a JEOL ECA-500 MHz NMR spectrometer operating at 500 MHz for ^1^H and 125 MHz for ^13^C (JEOL Ltd.) or an Agilent 700 MHz NMR spectrometer (Agilent Technologies, Inc.) equipped with a cryoprobe, operating at 700 MHz for ^1^H and 175 MHz for ^13^C. Residual solvent signals of CDCl_3_ (δH = 7.26 and δC = 77.0) were used for referencing.

**Encapsulated Nanodroplet Crystallization (ENaCt) and crystallographic details for 19-acetylstemphone G (1):** The crystallization of 19-acetylstemphone G (**1**) was carried out using encapsulated nanodroplet crystallization (ENaCt) protocols (Tyler et al., 2020). Stock solutions of **1** were prepared in methanol (4.0 mg in 1.2 mL), 100 µL of methanol stock solution aliquoted into 12x screw top glass vials. Solvent was allowed to evaporate in air, at room temperature, overnight, to give approximately 0.33 mg of 19-acetylstemphone G in each vial. Samples of **1** were then dissolved in a range of 12 different solvents (Dimethylsulfoxide (DMSO), *N*,*N*-dimethylformamide (DMF), methanol (MeOH), 1,1,1,3,3,3-hexafluoro-2-propanol (HFIP), toluene, 1,2-dichloroethane (1,2-DCE), 2-methyl tetrahydrofuran (2-Me THF), methyl *tert*-butyl ether (MTBE), ethyl acetate (EtOAc), acetonitrile (MeCN), 4-methylpentan-2-one (MIBK) and nitromethane (NM) (**Table S2**). The stock solutions of **1** (50 nL) were dispensed via an SPT Labtech Mosquito liquid handling robot into 96-well glass plates (SWISSCI LCP Modular, 100 mm spacer) containing either an appropriate crystallization oil (PDMSO, FC-40, FY and mineral oil; 200 nL) or no oil (**Table S3, Figure S10**). Plates were sealed with a glass cover slip and allowed to stand undisturbed at room temperature in the dark. After 14 days, plates were assessed visually and by cross-polarized light microscopy for crystal growth (Nikon SMZ1000, model C-DSS230 microscope, equipped with GXCAM-U3-5l digital camera). The results of the ENaCt experiments in each well were classified as: F = failed to dispense correctly due to liquid robot failure; 1 = still solvated; 2 = non-crystalline or amorphous material; 3 = microcrystals or small single crystals; 4 = large single crystals. From 288 individual ENaCt experiments, 43 wells (15 %) contained small single crystals (class 3) suitable for X-ray diffraction analysis. A single crystal of **1** grown from DMF (50 nL, *ca*. 28 mg/mL) encased in a droplet of mineral oil (Plate 1, D11) was analyzed by single crystal X-ray diffraction analysis (**Figure S11**).

Wells were opened with use of a tungsten carbide scriber to remove a small portion of the glass cover slide, and the crystal was manipulated using MiTeGen Kapton microtools. Crystals were transferred to a glass slide and extracted under oil (Fomblin YR-1800) and mounted onto a 35 µm MiTeGen Kapton loop. Single crystal X-ray diffraction data for **1** were collected on a Rigaku XtaLAB Synergy diffractometer using multilayer monochromated Cu Kα radiation (λ = 1.54180 Å), generated using a PhotonJet micro-focus sealed X-ray tube source and detected at a HyPix Arc-100, pixel array detector. Samples were flash-cooled to 150 K under N_2_ using an Oxford Cryosystems cryostream. Unit cell measurement, data collection and data reduction were performed using the software CrysAlisPRO (Rigaku, 2022). Numerical absorption correction was applied therein using Gaussian integration over a multi-faceted crystal model. The structure of **1** was solved using SHELXT (Sheldrick, 2015) and refined with SHELXL (Sheldrick, 2008) through the Olex2 interface (Dolomanov et al., 2009) (**Figure S12)**. Disorder in one of the DMF solvent molecules in **1** was treated by modelling the molecule over two sites, restraining the 1,2- and 1,3- distances of all non-hydrogen atoms in the two disorder components to similarity, then refining the chemical occupancies separately from the atomic displacement parameters (ADPs). It should be noted that absolute configuration cannot be determined by XRD alone due to the unreliable flack parameter, only relative stereochemistry can be inferred. Full details about the collection, solution and refinement of **1** are documented in CIF format, which have been deposited with the Cambridge Crystallographic Data Centre under CCDC 2303613. Crystallographic information for compound **1** are provided in **Table S4**.

**Cytotoxicity assays for confirmation of bioactivity of isolated compounds:** Bioactivity of compounds **1** and **2** were tested against human melanoma cancer cells (MDA-MB435) and human ovarian cancer cells (OVCAR3) that were procured from the American Type Culture Collection. We were unable to test compound **3** due to its rapid interconversion to the other analogs. Briefly, the cells were propagated at 37 °C in presence of 5% CO_2_ in RPMI 1640 medium, supplemented with fetal bovine serum (10%), penicillin (100 units/mL), and streptomycin (100 µg/mL). Cells in logarithmic growth phase were harvested by trypsinization followed by two washes to remove all traces of enzyme. A total of 5000 cells were seeded per well of a 96-well clear, flat-bottom plate (Microtest 96, Falcon) and incubated overnight at 37 °C in presence of 5% CO_2_. Samples dissolved in DMSO were then diluted and added to the appropriate wells (for a final concentration of 0.1% DMSO). The cells were incubated in the presence of the test substances for 72 h at 37 °C and evaluated for viability with a commercial absorbance assay (CellTiter 96^®^ AQ_ueous_ One Solution Cell Proliferation Assay, Promega Corp.) that measured viable cells. Survival percentage was expressed in percentage relative to the solvent (DMSO) control. Taxol (paclitaxel) was used as a positive control at a concentration of 10 nM. Compounds **1-2** were analyzed in 3 biological replicates with 3 technical replicates each time, at concentrations ranging from 8 nM - 25 μM.

**Confirmation of endogenous metabolites using microextraction-enabled droplet probe mass spectrometry:** To confirm that the acetylation of the new stemphone analogs was not an artefact of our extraction/fractionation process, droplet probe studies were conducted with Petri dishes of the three *Aspergillus* strains used in this study. Due to the production of spores by *Aspergillus* *spp.* the mycelia were removed from the petri dishes prior to examination and the agar surrounding the mycelia were analyzed (**Figures S13-15**) using a converted CTC/LEAP HTC PAL autosampler (LEAP Technologies Inc.) as detailed previously (Cank et al., 2021; Sica et al., 2015). Briefly, microextractions were performed using Fisher Optima LC/MS grade solvents consisting of 50:50 methanol: water. An initial 5 µL of solvent were drawn into the syringe. Droplets of 4 µL were dispensed onto the surface of the sample at a rate of 2 µL/s, held on the surface for 2 seconds, and withdrawn back into the syringe at the same rate. This extraction process was repeated a total of three times for a single spot prior to injection into the UPLC-MS system, which included an Acquity UPLC system (Waters Corp.) coupled to a Thermo Fisher Scientific Q Exactive Plus mass spectrometer. Standards were produced by depositing the three isolated compounds onto glass microscope slides and then performing a microextraction with the droplet probe to obtain their retention time on the column (**Figure S16**). All three compounds were observed in the agar indicating that they were produced as elucidated by the fungus as endogenous metabolites.

# **Table S1.** MZMine Processing Parameters.

| **1.** | **Mass Detection** | | |  |
| --- | --- | --- | --- | --- |
|  | Noise level MS^1^ | | 5E6 |  |
|  | Noise level MS^2^ | | NA |  |
| **2.** | **Peak detection -> ADAP chromatogram builder** | | |  |
|  | Min group size in # of scans | | 3 |  |
|  | Group intensity threshold | | 5E6 |  |
|  | Min highest intensity | | 1E6 |  |
|  | *m/z* tolerance | | 0.001 m/z OR 5 ppm |  |
| **3.** | **Peak detection -> Chromatogram deconvolution wavelets (ADAP)** | | |  |
|  | *m/z* center calculation | | MEDIAN |  |
|  | S/N threshold | | 10 |  |
|  | S/N estimator: | | intensity window SN |  |
|  | Min feature height | | 1E6 |  |
|  | Coefficient area threshold | | 100 |  |
|  | Peak duration range [min] | | 0.01 - 0.5 |  |
|  | RT wavelet range [min] | | 0.00 - 0.1 |  |
| **4.** | **Isotopic peaks grouper** | | |  |
|  | *m/z* tolerance | | 0.01 *m/z* |  |
|  | Retention time tolerance [min] | | 0.05 |  |
|  | Representative isotope | | Most Intense |  |
| **5.** | **Join Aligner** | | |  |
|  | *m/z* tolerance | | 0.001 m/z OR 7 ppm |  |
|  | Weight for *m/z* | | 10 |  |
|  | Retention time tolerance [min] | | 0.15 |  |
|  | Weight for Retention time | | 10 |  |
|  | Require same charge state on | | **✓ ON** |  |
| **6.** | **Filter Peak List by Row** | | |  |
|  | Minimum peaks in a row | | 2 |  |
|  | *m/z* values | | 150-1500 |  |
|  | Retention time range [min] | | 0.25 – 9.00 |  |
|  | Reset Peak number ID | | **✓** |  |
|  | Keep only peaks with MS^2^ scan | | **✓** |  |
| **7.** | **Gap Filling** | | |  |
|  | Intensity tolerance | | 0.2 |  |
|  | *m/z* tolerance | | 0.001 m/z OR 7 ppm |  |
|  | Retention time tolerance [min] | | 0.15 |  |
| **8.** | **Export** | | |  |
|  | Export to .csv | | **✓** |  |
|  | Export to .mgf | | **✓** |  |
|  | Merge MS/MS (experimental): | | |  |
|  | | Select spectra to merge | Across Samples |  |
|  | | *m/z* merge mode | Weighted Average |  |
|  | | Intensity merge mode | Sum Intensities |  |
|  | | Expected mass deviation | 0.001 m/z OR 10.0 ppm |  |
|  | | Cosine threshold (%) | 70 |  |
|  | | Peak count threshold (%) | 20 |  |
|  | | Isolation offset window | 0.000 |  |
|  | | Isolation window width | 3.00 |  |


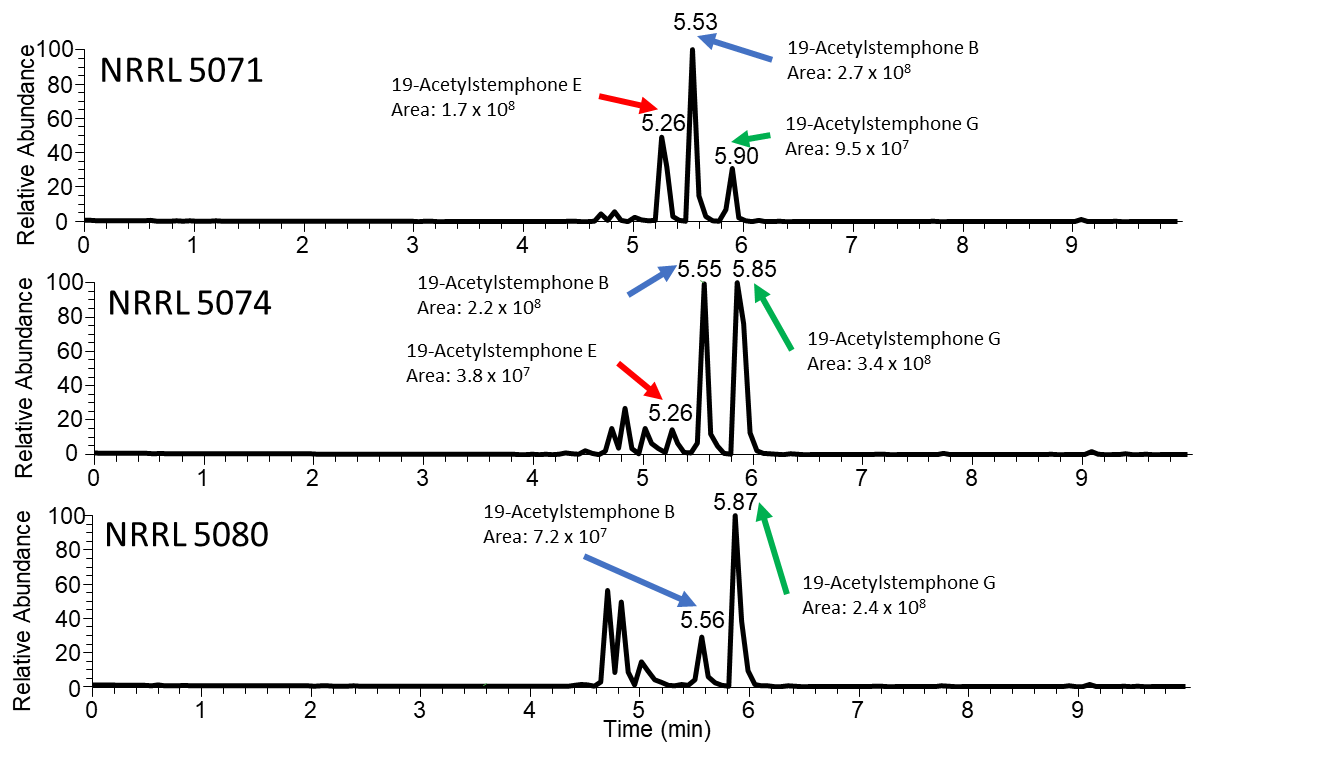


# **Figure S1.** UPLC chromatograms of flash chromatography fractions from NRRL 5071, 5074, and 5080.

X

X

# **Figure S2**. 1H and 13C NMR spectra of 19-aceytlstemphone G (compound **1**; 700 MHz, CDCl_3_).

# **Figure S3.** COSY NMR spectrum of 19-acetylstemphone G (compound **1**; 700 MHz, CDCl_3_).

# **Figure S4**. HSQC NMR spectrum of 19-acetylstemphone G (compound **1**; 700 MHz, CDCl_3_).

# **Figure S5**. HMBC NMR spectrum of 19-acetylstemphone G (compound **1**; 700 MHz, CDCl_3_).

# **Figure S6.** NOESY NMR spectrum of 19-acetylstemphone G (compound **1**; 700 MHz, CDCl_3_).

# **Figure S7.** Key HMBC correlations for 19-acetylstemphone G (compound **1**).

Connection of the olefinic side chain to the aromatic ring was confirmed by correlations from H3-27 and H-4 to C-6 (δc140.4), while the terpenoid portion was confirmed by key HMBC correlations between H3-23 and H3-24 to an oxygenated quaternary carbon C-22 (δc71.7) as well as to oxygenated methine C-21 (δc80.0). H2-20 displayed HMBC correlations to C-21 and C-22, confirming the placement of the isopropanol side chain, and had correlations to oxygenated methine C-19 (δc73.1) and quaternary carbon C-18 (δc38.6). Methyl H3-25 showed HMBC correlations to C-18, C-19, and oxygenated methine C-17 (δc76.4). H-17 showed HMBC correlations to C-21, through the oxygen to which they are both connected, and to C-19 and C-25, which completes the C-17 to C-21 heterocycle. All that remained was to connect the two pieces. Thus, the last two rings were established using HMBC correlations between H-13 and C-12, C-17, C-18, C-19, and C-25, which connected the heterocycle to the α,β-unsaturated ketone. Correlations between H-13 and oxygenated quaternary carbon C-14 (δc83.7) and methyl C-26 (δc22.1) completed the six membered heterocycle fused to the aromatic ring. The last ring was closed with HMBC correlations observed between H-17 and C-16 (δc24.7) and between H2-15 and C-13, C-16, and C-26.

# **Figure S8.** 1H NMR spectrum of 19-acetylstemphone B (compound **2**; 400 MHz, CDCl_3_).

# **Figure S9.** 1H NMR spectrum of 19-acetylstemphone E (compound **3**; 400 MHz, CDCl_3_).

# **Table S2.** Preparation of stock solutions of 19-acetylstemphone G (compound **1**) for ENaCt experiments.

| **Vial** | **Solvent** | **Mass of compound (mg)** | **Solvent Volume (µL)** | **Concentration (mg/mL)** |
| --- | --- | --- | --- | --- |
| 1 | DMSO | 0.33 | 12 | 27.5 |
| 2 | DMF | 0.33 | 12 | 27.5 |
| 3 | MeOH | 0.33 | 12 | 27.5 |
| 4 | HFIP | 0.33 | 12 | 27.5 |
| 5 | Toluene | 0.33 | 12 | 27.5 |
| 6 | DCE | 0.33 | 12 | 27.5 |
| 7 | THF | 0.33 | 12 | 27.5 |
| 8 | MTBE | 0.33 | 12 | 27.5 |
| 9 | EtOAc | 0.33 | 12 | 27.5 |
| 10 | MeCN | 0.33 | 12 | 27.5 |
| 11 | MIBK | 0.33 | 12 | 27.5 |
| 12 | NM | 0.33 | 12 | 27.5 |

**Table S3.** Encapsulation oils for ENaCt Experiments.

| **ENaCt Oils** | |
| --- | --- |
| PDMSO | poly(dimethylsiloxane); CAS: 63148-62-9; supplier: Sigma Aldrich |
| FC-40 | Fluorinert FC-40; CAS: 51142-49-5; supplier: Fluorochem |
| FY | Fomblin YL VAC; CAS: 69991-67-9; supplier: Solvay |
| MO | Mineral oil; CAS: 8042-47-5; supplier: Sigma Aldrich |

# **Figure S10.** Plate setup and crystallization results for the ENaCt protocol used, detailing the type, amount and concentration of the respective stock solution and oil as well as the classification of the wells after two weeks.

Key: F = failed to dispense correctly due to liquid robot failure; 1 = still solvated; 2 = non-crystalline or amorphous material; 3 = microcrystals or small single crystals; 4 = large single crystals.

**
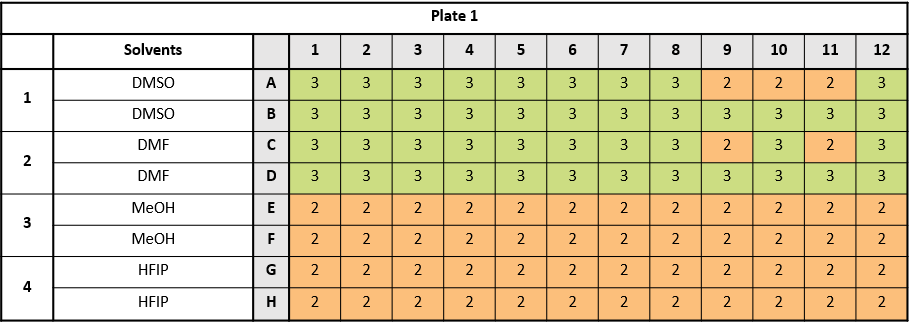
**
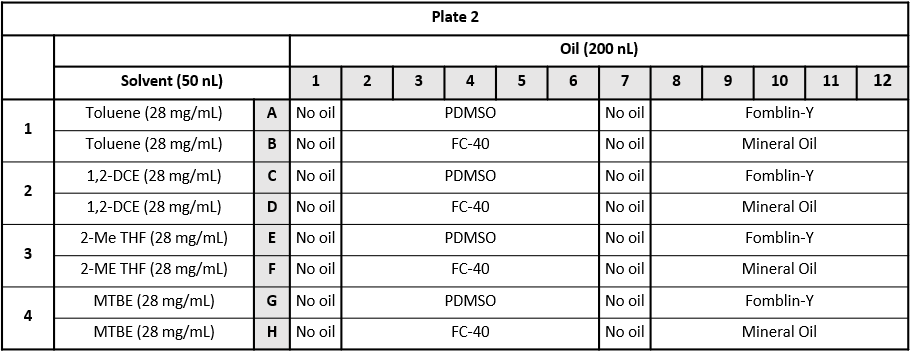

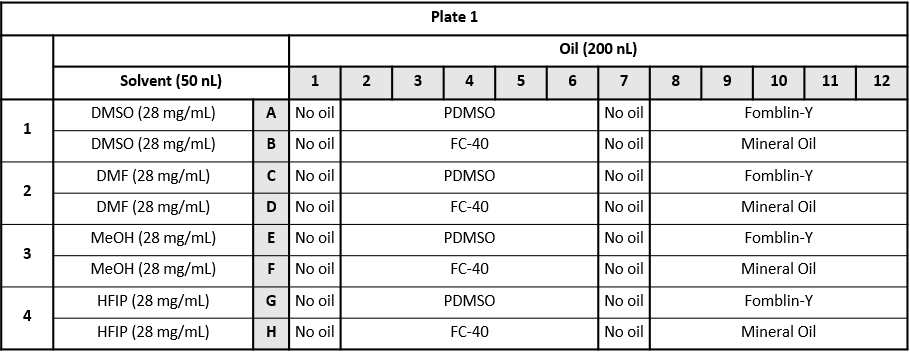


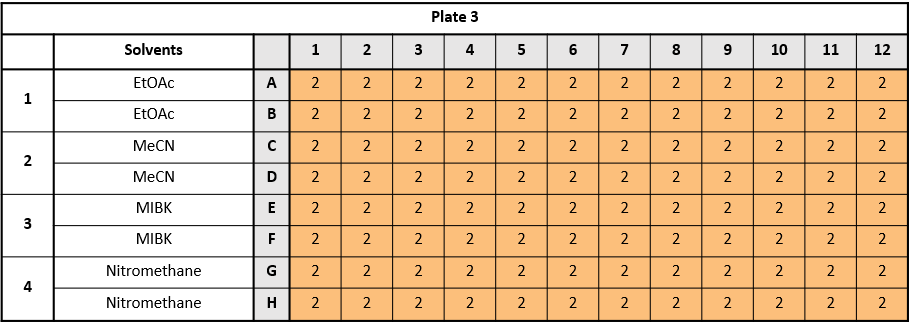

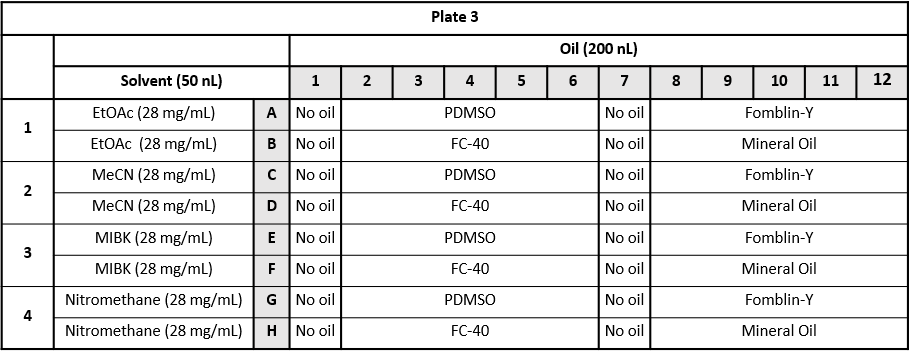

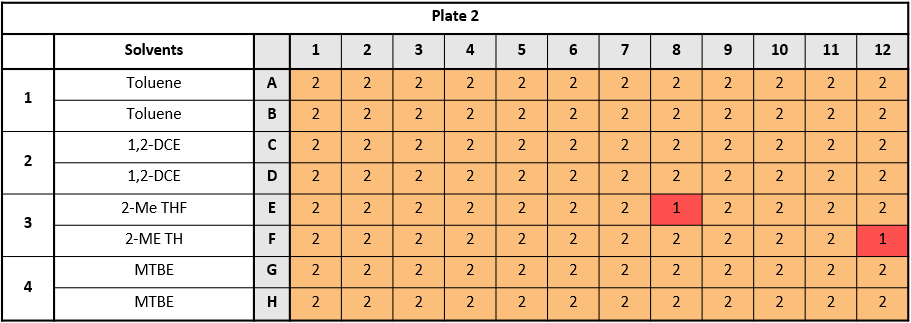


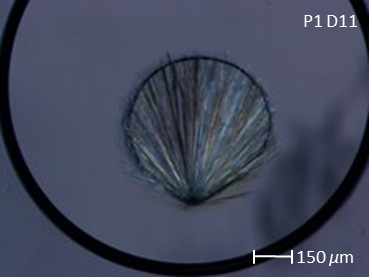


# **Figure S11.** An image of Plate 1 well D11, with a scale bar.


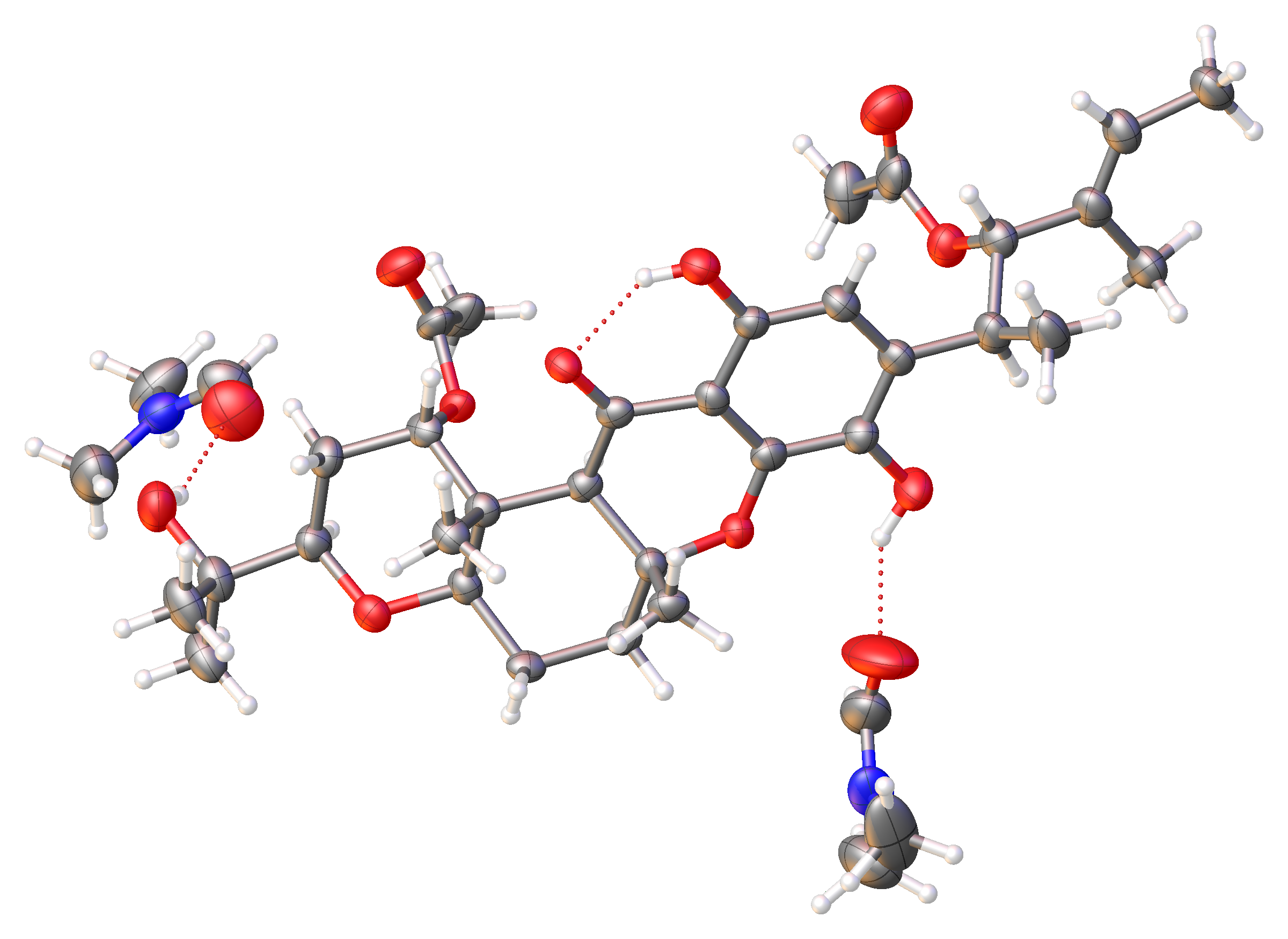


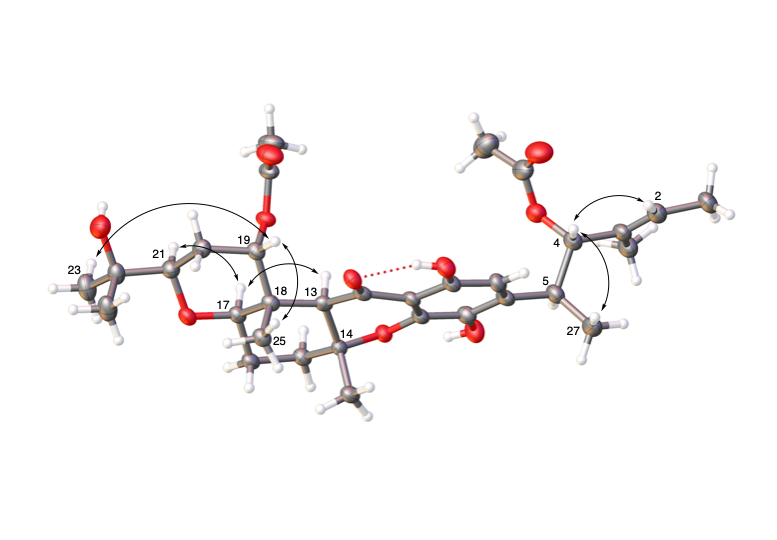


# **Figure S12.** X-ray crystallographic structure 19-acetylstemphone G (compound **1**), showing DMF molecules within the structure (top) and overlaid with key NOESY correlations (bottom).

# **Table S4.** Crystallographic parameters for compound **1**.

| Identification code | MRP_AL_SG |
| --- | --- |
| CCDC code | CCDC 2303613 |
| Empirical formula | C_38_H_58_N_2_O_12_ |
| Formula weight | 367.43 |
| Temperature/K | 150.00(10) |
| Crystal system | monoclinic |
| Space group | *P*2_1_ |
| *a*/Å | 6.2552(3) |
| *b*/Å | 15.0915(10) |
| *c*/Å | 20.5773(9) |
| *α*/° | 90 |
| *β*/° | 96.552(5) |
| *γ*/° | 90 |
| Volume/Å^3^ | 1929.82(18) |
| *Z* | 4 |
| *ρ*_calc_g/cm^3^ | 1.265 |
| *μ*/mm^‑1^ | 0.772 |
| F(000) | 792.0 |
| Crystal size/mm^3^ | 0.234 × 0.022 × 0.021 |
| Radiation | Cu Kα (λ = 1.54184) |
| 2Θ range for data collection/° | 7.28 to 157.54 |
| Index ranges | -7 ≤ h ≤ 3, -19 ≤ k ≤ 17, -24 ≤ l ≤ 26 |
| Reflections collected | 14285 |
| Independent reflections | 6237 [R_int_ = 0.0875, R_sigma_ = 0.1097] |
| Data/restraints/parameters | 6237/566/538 |
| Goodness-of-fit on F^2^ | 1.045 |
| Final R indexes [I>=2σ (I)] | R_1_ = 0.0763, wR_2_ = 0.1773 |
| Final R indexes [all data] | R_1_ = 0.1220, wR_2_ = 0.2017 |
| Largest diff. peak/hole / e Å^-3^ | 0.57/-0.29 |
| Flack parameter | 0.3(3) |


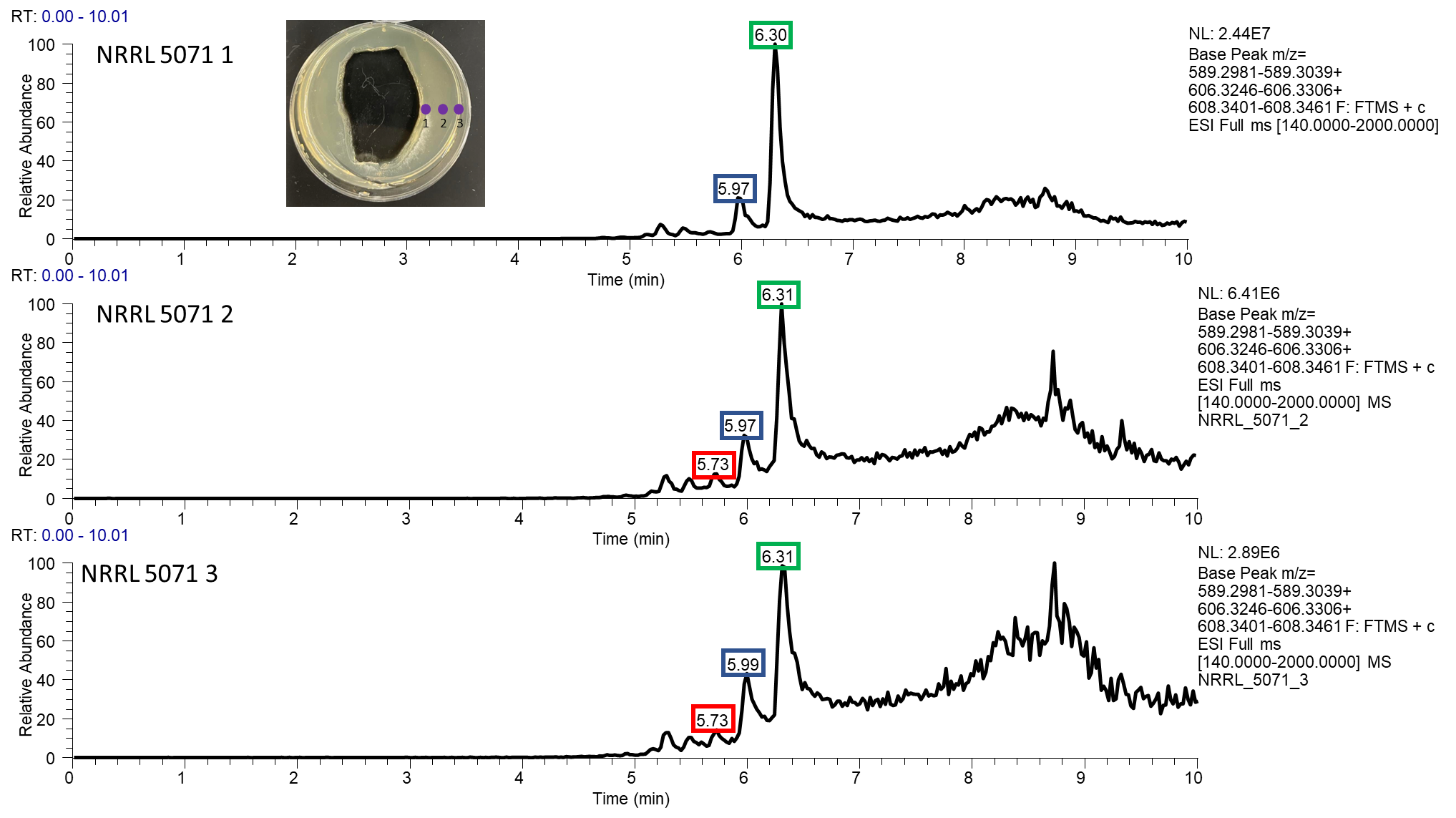


# **Figure S13.** UPLC chromatograms from droplet probe analysis of NRRL 5071.


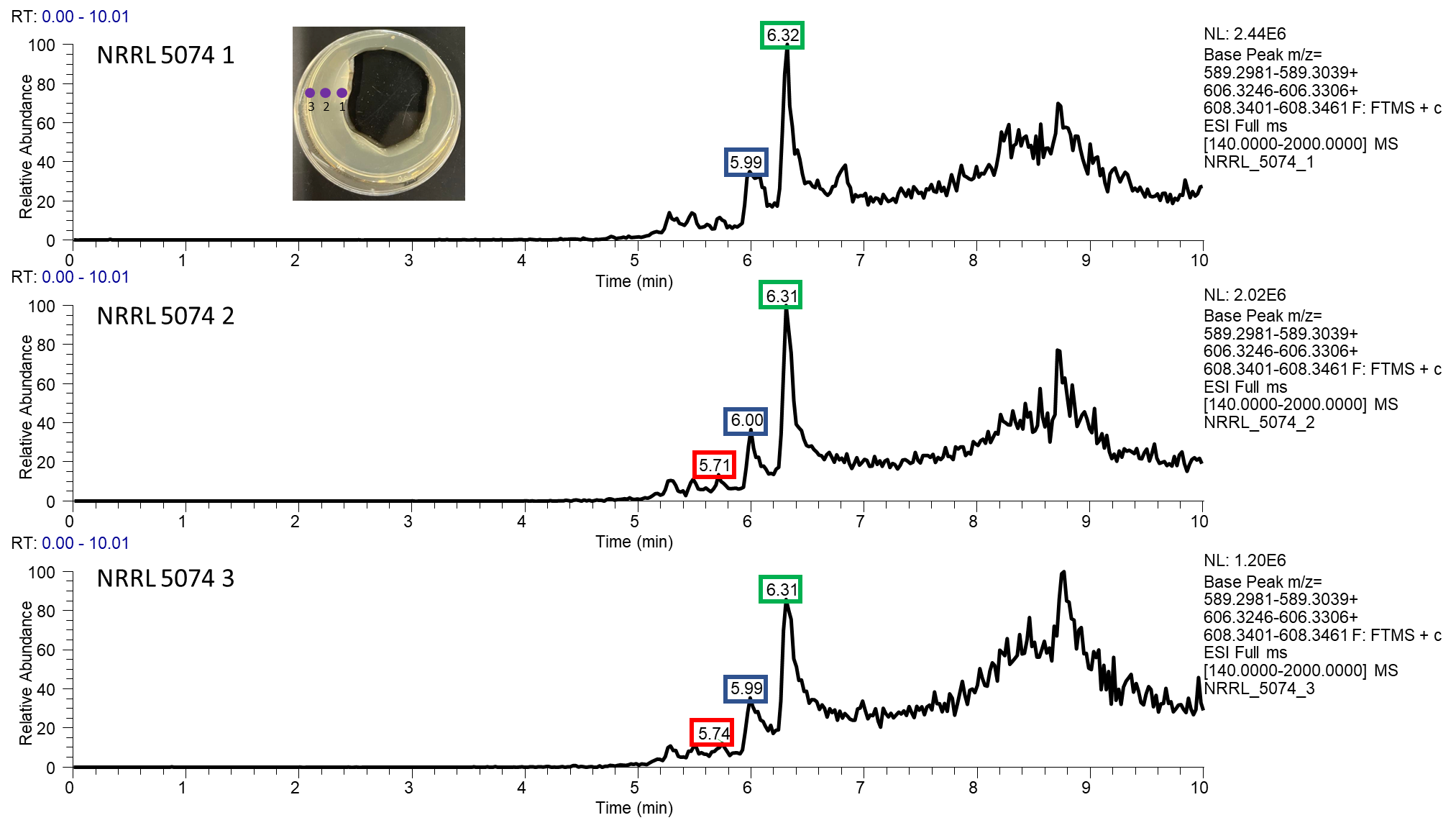


# **Figure S14.** UPLC chromatograms from droplet probe analysis of NRRL 5074.


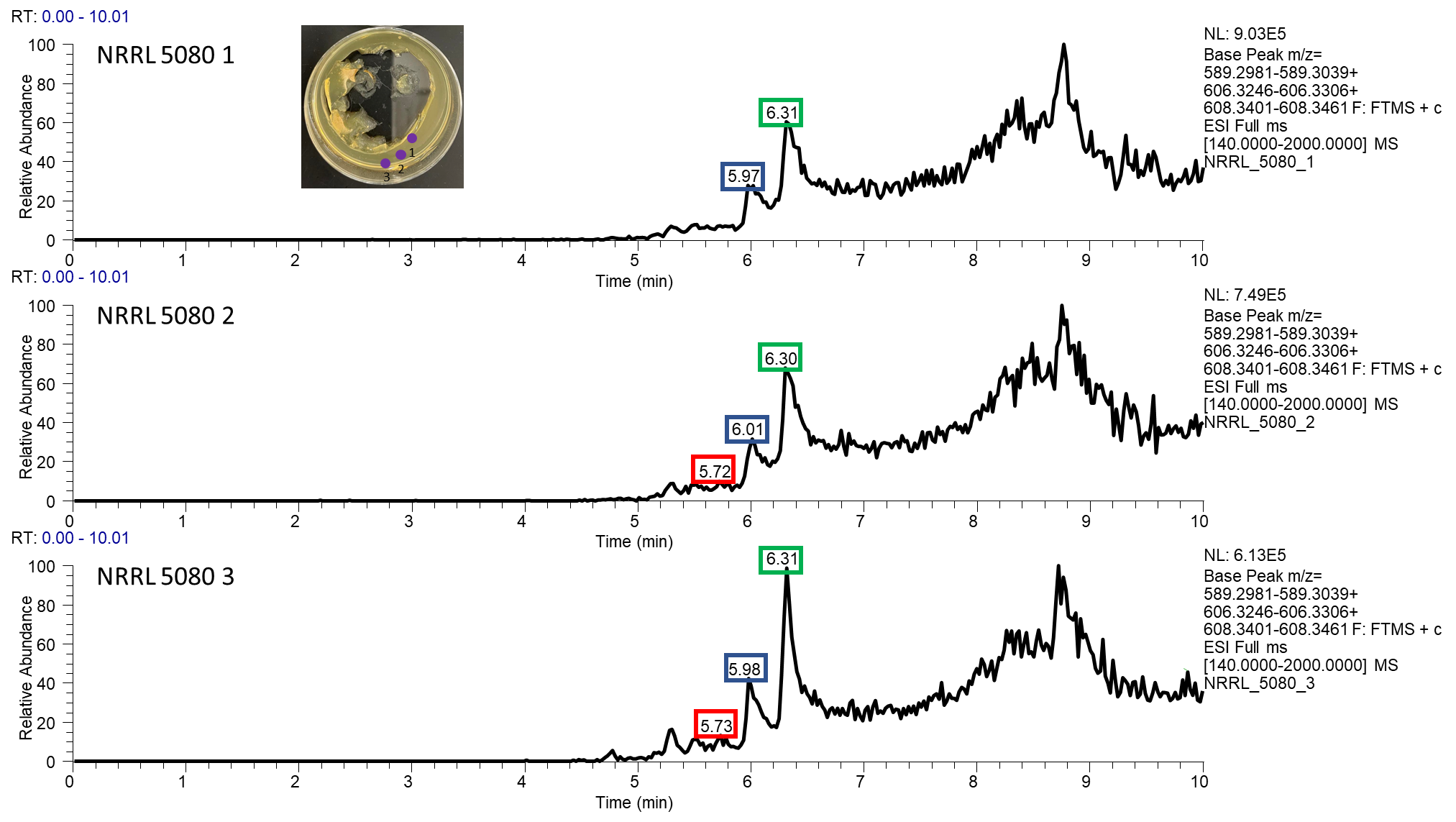


# **Figure S15.** UPLC chromatograms from droplet probe analysis of NRRL 5080.


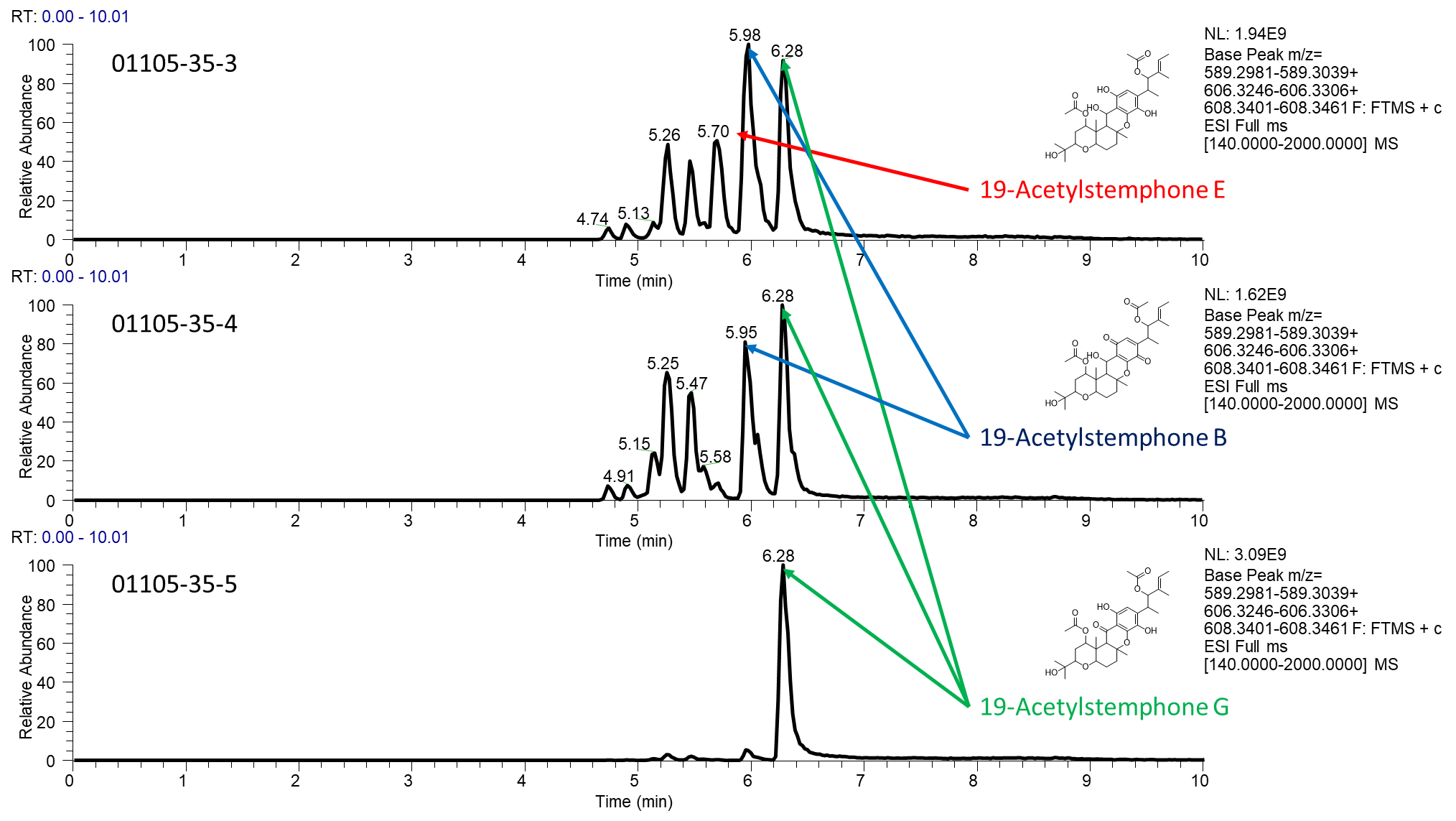


# **Figure S16.** UPLC chromatograms from droplet probe analysis of standards of compounds **1**, **2**, and **3**.

Note that in 01105-35-3 and 01105-35-4, numerous degradation products exist potentially due to rearrangement from introduction of methanol/water. Additionally, 19-acetylstemphone E and B both eventually convert to 19-acetylstemphone G. These experiments were conducted a few months after structure elucidation, so standards had begun to degrade.


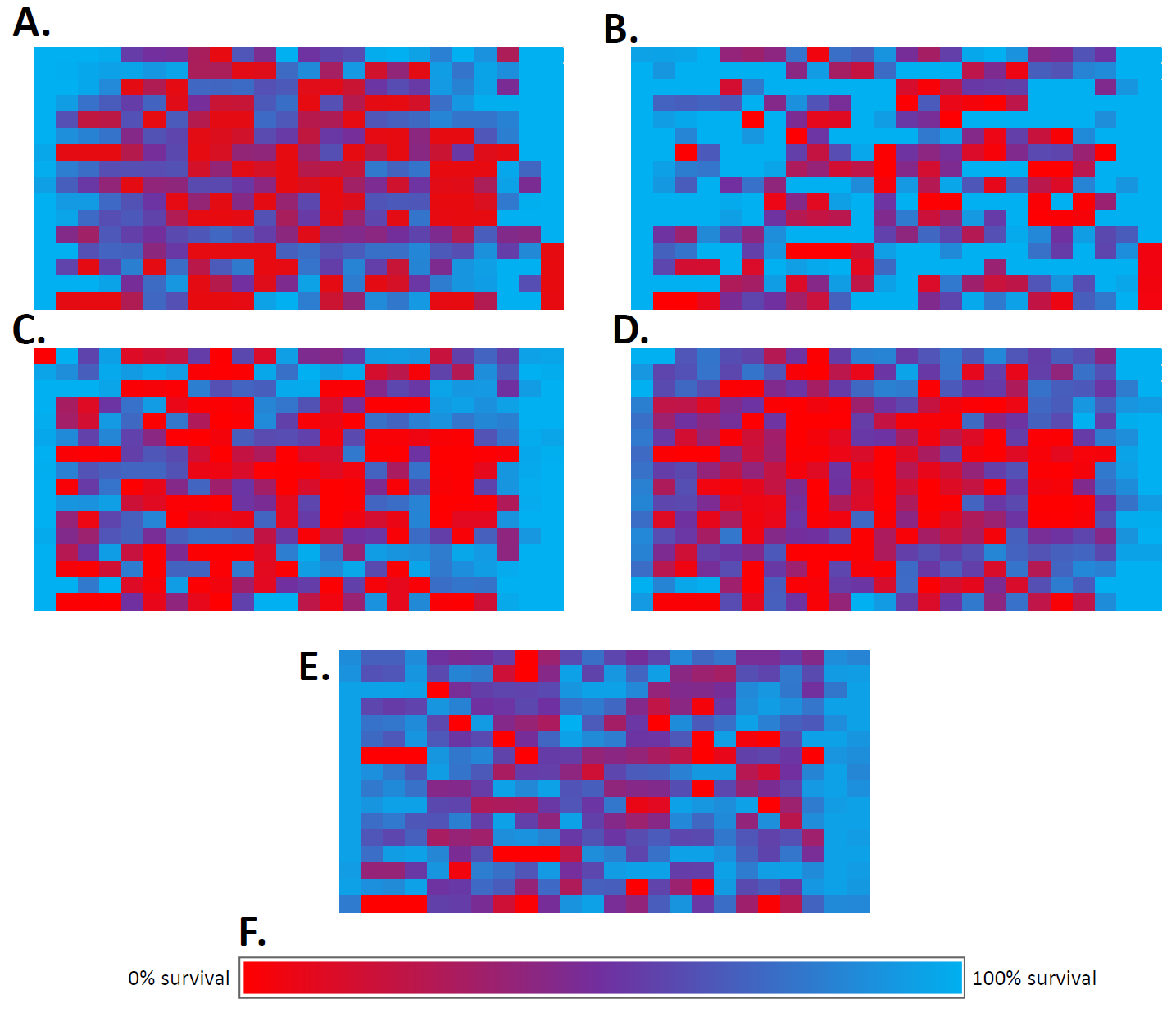


**Figure S17.** Heatmaps of secondary high-throughput screening of fungal strains against (A) MCF10a human mammary epithelial, (B) A549 lung cancer, (C) HCT 116 colon cancer, (D) LN229 glioblastoma, and (E) MCF7 breast cancer cell lines. Panel (F) shows the color scheme for the heat maps, ranging from 0-100% survival. In instances where the percent survival was measured to be greater than 100% (based on the average of the blanks), values were normalized to 100%. Extracts were randomly placed on plates at 3 concentrations in triplicate. The associated plate layout can be found in Online Resource 1.


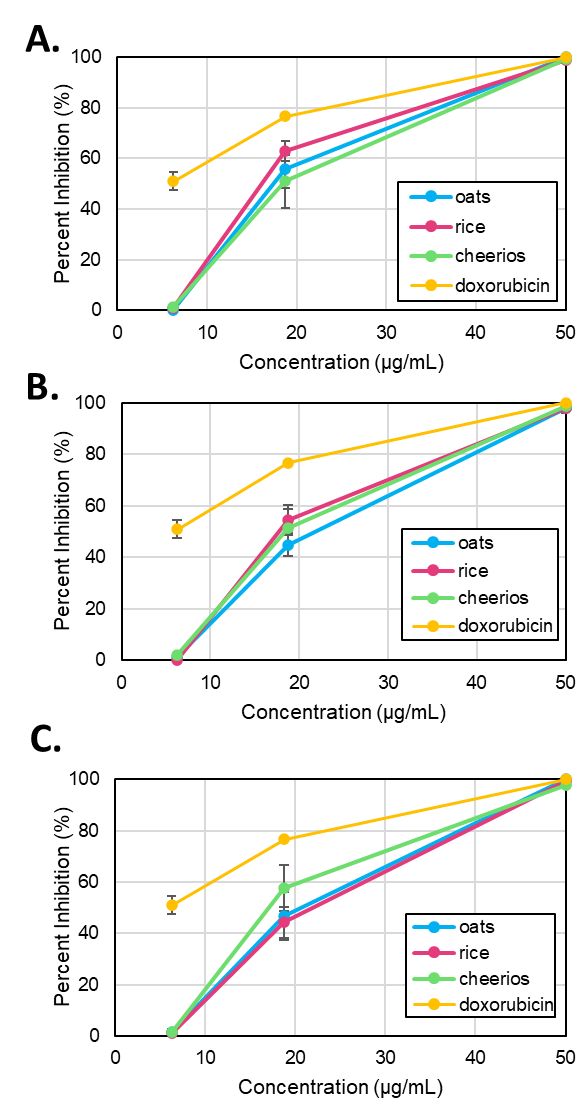


# **Figure S18.** Dose response curves of (A) NRRL 5071, (B) NRRL 5074, and (C) NRRL 5080 against MCF-7 cells.


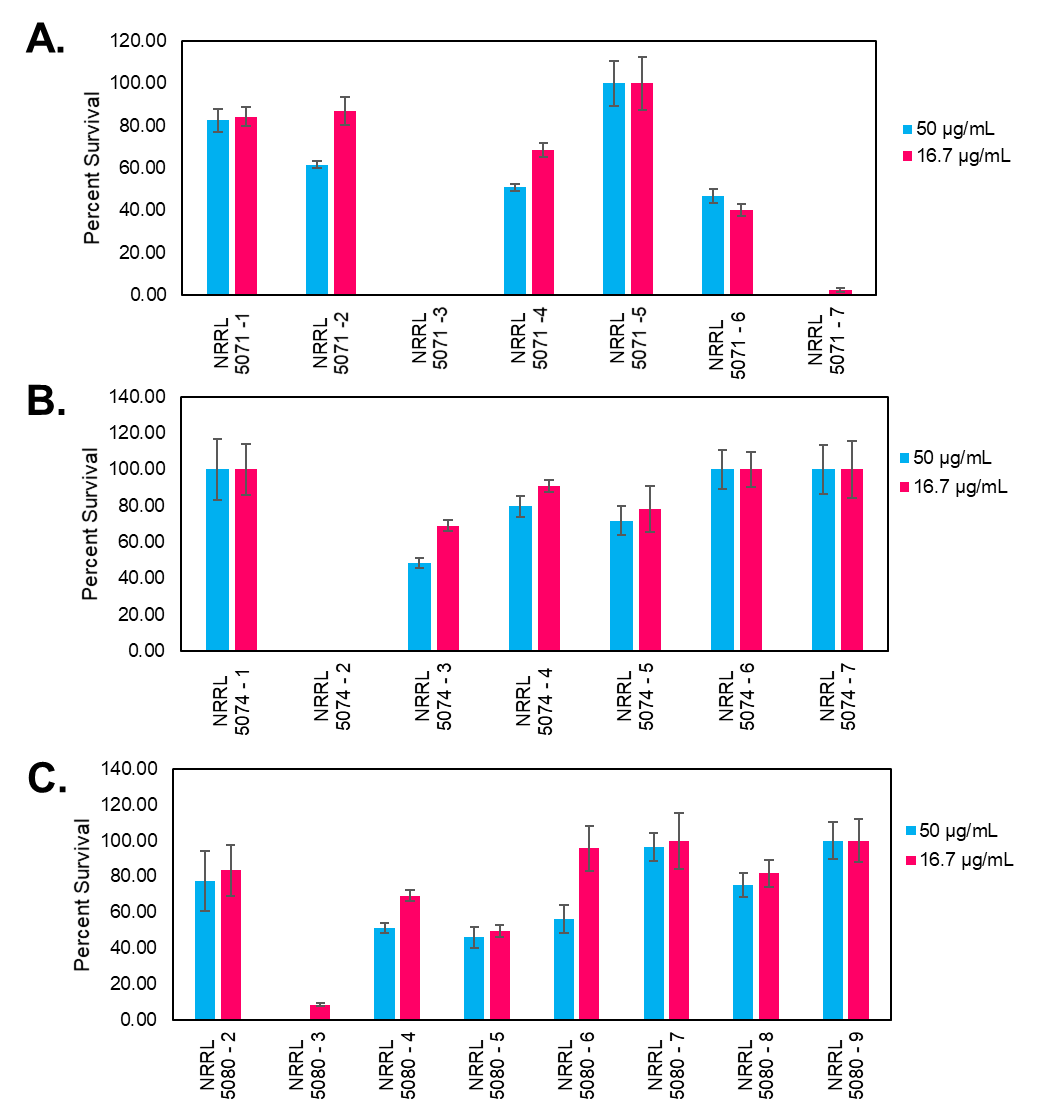


# **Figure S19.** Bioactivity of flash chromatography fractions from (A) NRRL 5071, (B) NRRL 5074, and (C) NRRL 5080 against MCF-7 cells used for biochemometrics analysis.

# **Figure S20**. Comparison of the experimental and calculated ECD spectra for 4S, 5S, 13R, 14R, 17R, 18R, 19S, 21R 19-acetylstemphone G (compound **1**).

# **Table S5.** Antiproliferative/cytotoxicity data for compounds **1** and **2** against OVCAR3 and MDA-MB-435 cell lines.

|  | IC_50_ (µM) in OVCAR3 | IC_50_ (µM) in MDA-MB-435 |
| --- | --- | --- |
| Compound **1** | 4.53 ± 0.10 | 4.40 ± 0.10 |
| Compound **2** | 3.58 ± 0.24 | 4.20 ± 0.02 |

# **Table S6:** Correlation scores for the 19-acetylstemphones with biosynthetic gene cluster families.

| **Correlation Score (Rank)^a^** | **PKS-containing Gene Cluster Families^b^** | | | | | | | | | | | | | | | | | |
| --- | --- | --- | --- | --- | --- | --- | --- | --- | --- | --- | --- | --- | --- | --- | --- | --- | --- | --- |
|  | **PRPKS Gene Cluster Families** | | | | **HRPKS Gene Cluster Families** | | | | | | | | | **NRPKS Gene Cluster Families** | | | | |
|  | 244 | 263 | 265 | 266 | 322 | 363 | 365 | 471 | 472 | 473 | 475 | 476 | 477 | 110 | 208 | 272 | 274 | 276 |
| Compound **1** 19-acetylstemphone G | 125 (1) | 106 (4) | 106 (4) | 106 (4) | 104 (5) | 125 (1) | 104 (5) | 106 (4) | 106 (4) | 106 (4) | 106 (4) | 106 (4) | 106 (4) | 123 (3) | 124 (2) | 106 (4) | 106 (4) | 106 (4) |
| Compound **2**  19-acetylstemphone B | 136 (1) | 117 (6) | 117 (6) | 117 (6) | 115 (5) | 136 (1) | 115 (5) | 117 (6) | 117 (6) | 117 (6) | 117 (6) | 117 (6) | 117 (6) | 134 (3) | 135 (2) | 117 (6) | 117 (6) | 117 (6) |
| Compound **3**  19-acetylstemphone E | 125 (1) | 106 (4) | 106 (4) | 106 (4) | 104 (5) | 125 (1) | 104 (5) | 106 (4) | 106 (4) | 106 (4) | 106 (4) | 106 (4) | 106 (4) | 123 (3) | 124 (2) | 106 (4) | 106 (4) | 106 (4) |

^a^Note that several compound-metabolite pairs shared the same correlation score ranking. PRPKS_244 and HRPKS_363 were consistently tied for first rank across all three compounds of interest

^b^Gene cluster families are named by BiosyntheticType_IdentifyingNumber (*e.g.* PRPKS_244). The numbers indicated here refer to the identifying number for PKS-containing GCFs. All GCFs were grouped as part of a previous project (Caesar et al., 2023).


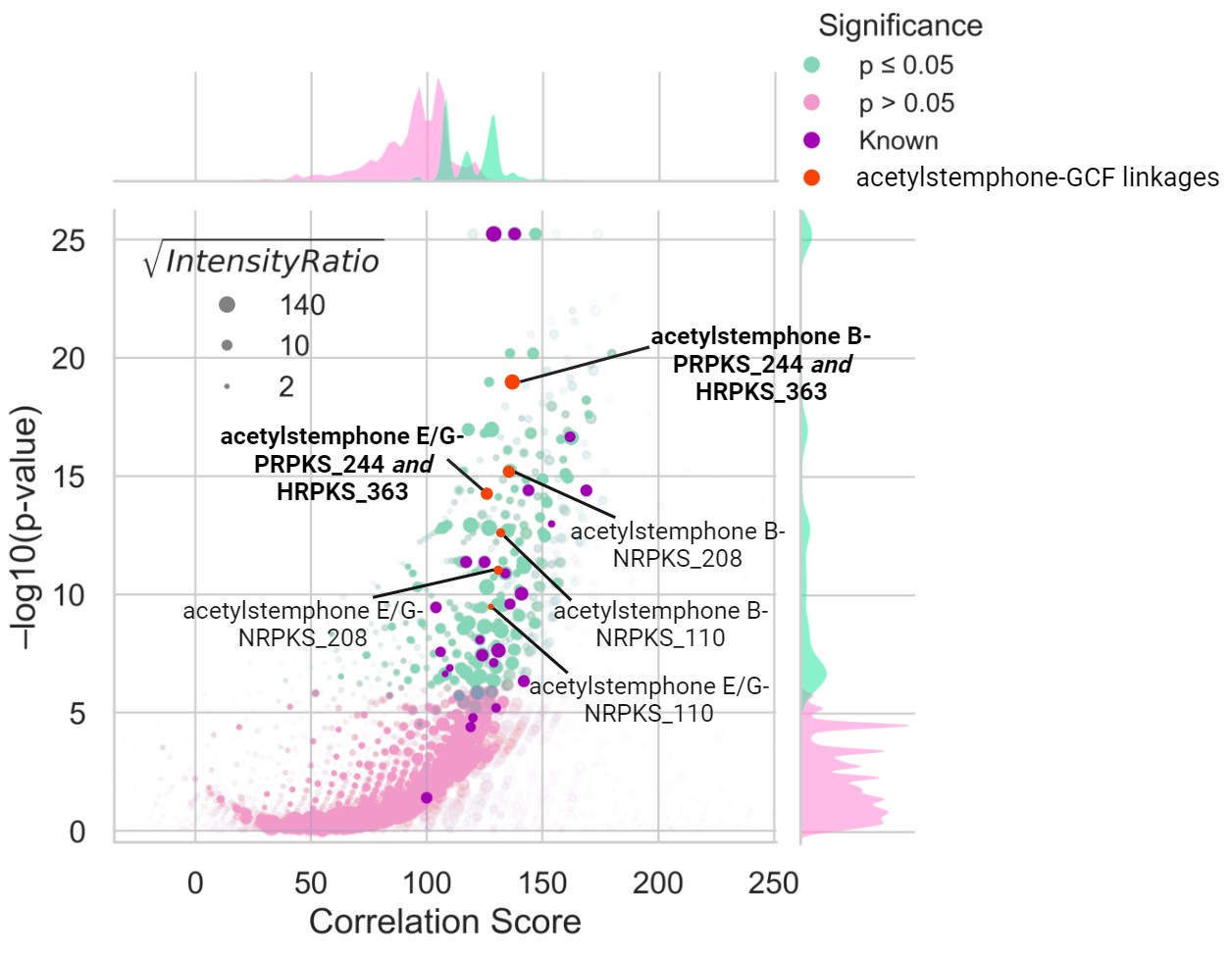


# **Figure S21.** Compiled metabolite-GCF correlations for acetylstemphones.

Each point represents a unique metabolite–GCF pair whose location corresponds to the strength of the association. Weighted correlation scores are on the x axis and −log10(P values) on the y axis. P values are the result of a chi-squared test with a Bonferroni correction. Significant correlations (P ≤ 0.05 after multiple-hypothesis correction) and nonsignificant correlations are colored in green and pink, respectively. Correlations for validated metabolite–GCF pairs are in purple and correlations between acetylstemphones and four high-ranking PKS-containing GCFs of interest are colored in orange.

**Figure S22.** Structurally-related meroterpenoids sharing a 6/6/6/6-tetracyclic ring system consisting of sesquiterpenoid and polyketide components

# **Table S7.** Comparative analysis of stm gene cluster and atn gene cluster.

| ***Arthrinium* sp. NF2194** | | | ***Aspergillus biplanus* NRRL 5071** | | **Putative function** | **Sequence Homologous** |
| --- | --- | --- | --- | --- | --- | --- |
| **Gene** | **Accession** | **Size** | **Gene** | **Size** |  |  |
| atnA | MH183007 | 751 | stmJ | 474 | FAD-dependent monooxygenase | 27.54% |
| atnC | MH183009 | 435 | stmH | 417 | acetyltransferase | 35.86% |
| atnF | MH183012 | 312 | stmA | 316 | polyprenyl transferase | 31.58% |
| atnG | MH183013 | 1985 | stmE | 1773 | NR-PKS | 26.37% |
| atnH | MH183014 | 2466 | stmK | 2466 | PR-PKS | 31.13% |
| atnI | MH183015 | 707 | stmS | 688 | terpene cyclase | 23.31% |
| atnK | MH183017 | 756 | stmC | 456 | FAD-dependent monooxygenase | 40.09% |
| atnM | MH183019 | 480 | stmD | 537 | P450 | 32.36% |

**
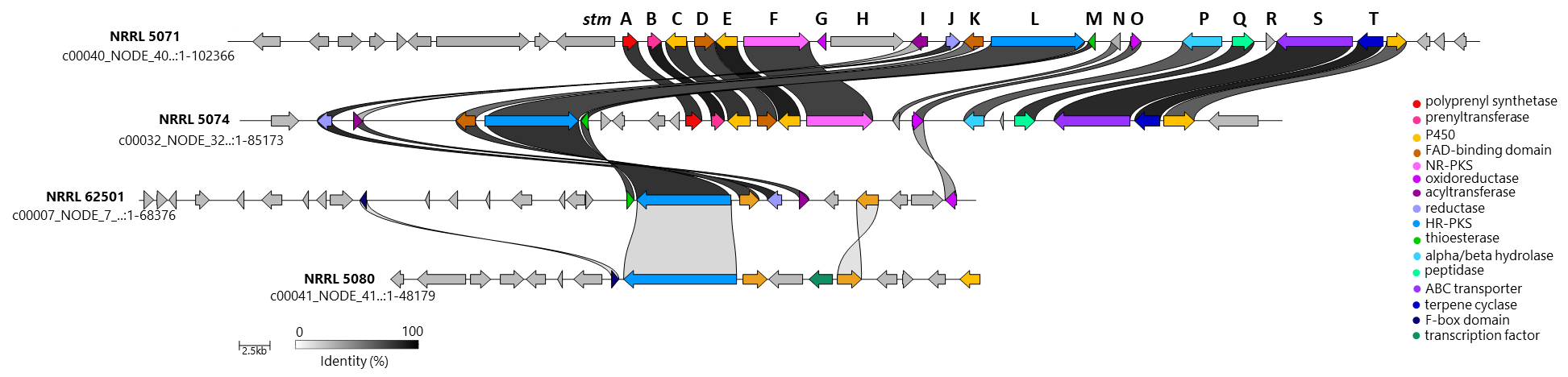
**

# **Figure S23.** Schematic representation of the members of the PRPKS_244 gene cluster family and the amino acid sequence identities.

# **Table S8.** Annotation of the *stm* gene cluster from *Aspergillus biplanus* NRRL 5071.

| **Gene** | **Size (aa)** | **Homologous protein** | **Plausible Function** | **Identity** |
| --- | --- | --- | --- | --- |
| Downstream gene A | 301 | hypothetical protein (KAJ5554678.1) | hypothetical | 52.23% |
| Downstream gene B | 1007 | O-methylsterigmatocystin oxidoreductase (XP_056559663.1) | oxidoreductase | 57.60% |
| stmA | 365 | Geranylgeranyl pyrophosphate synthetase (KIA75413.1) | polyprenyl synthetase | 79.36% |
| stmB | 316 | Cle5 *Aspergillus versicolor* (BBG28475.1) | prenyltransferase | 74.53% |
| stmC | 482 | Cle4 *Aspergillus versicolor* (BBG28474.1) | cytochrome P450 monooxygenase | 73.93% |
| stmD | 456 | Cle3 *Aspergilus versicolor* (BB73.1)G284 | flavin-dependent monooxygenase | 70.00% |
| stmE | 537 | Cle2 *Aspergilus versicolor* (BBG24872.1) | cytochrome P450 monooxygenase | 82.06% |
| stmF | 1773 | Cle1 *Aspergilus versicolor* (BBG28471.1) | polyketide synthase | 60.12% |
| stmG | 204 | oxidoreductase (XP_056919798) | dioxygenase | 64.32% |
| stmH | 655 | hypothetical protein (KAJ5701956) | hypothetical | 67.53% |
| stmI | 417 | atnC (A0A455LM26.1) | acyl-transferase | 35.86% |
| stmJ | 271 | hypothetical protein (KKK19914.1) | hypothetical | 56.27% |
| stmK | 474 | FAD binding domain protein *Talaromyces islandicus* (CRG87305.1) | FAD binding domain protein | 40.09% |
| stmL | 2506 | polyketide synthase *Aspergilus stellatus* (BBI47418.1) | polyketide synthase | 57.28% |
| stmM | 190 | thioesterase family (RJE26767.1) | thioesterase | 37.17% |
| stmN | 232 | uncharacterized protein (XP_056738422) | hypothetical | 61.97% |
| stmO | 273 | peptidyl-tRNA hydrolase 2 (XP_056738421.1) | hydrolase | 54.43% |
| stmP | 484 | alpha/beta hydrolase (KAH8816532.1) | alpha-beta hydrolase | 60.52% |
| stmQ | 487 | peptidase S28 (XP_051308759.1) | peptidase | 34.17% |
| stmR | 218 | hypothetical protein (KAH8812871.1) | hypothetical | 62.16% |
| stmS | 1767 | ATP-binding cassette transporter (XP-015404225.1) | ABC transporter | 62.81% |
| stmT | 688 | squalene-hopene-cyclase (XP-015404223.1) | terpene cyclase | 73.16% |
| Upstream gene A | 484 | alkane hydroxylase (XP_015404224) | hydroxylase | 70.47% |
| Upstream gene B | 308 | hypothetical protein (XP_0570088251.1) | hypothetical | 66.01% |
| Upstream gene C | 277 | Hypothetical protein (PYI08397.1) | hypothetical | 70.04% |


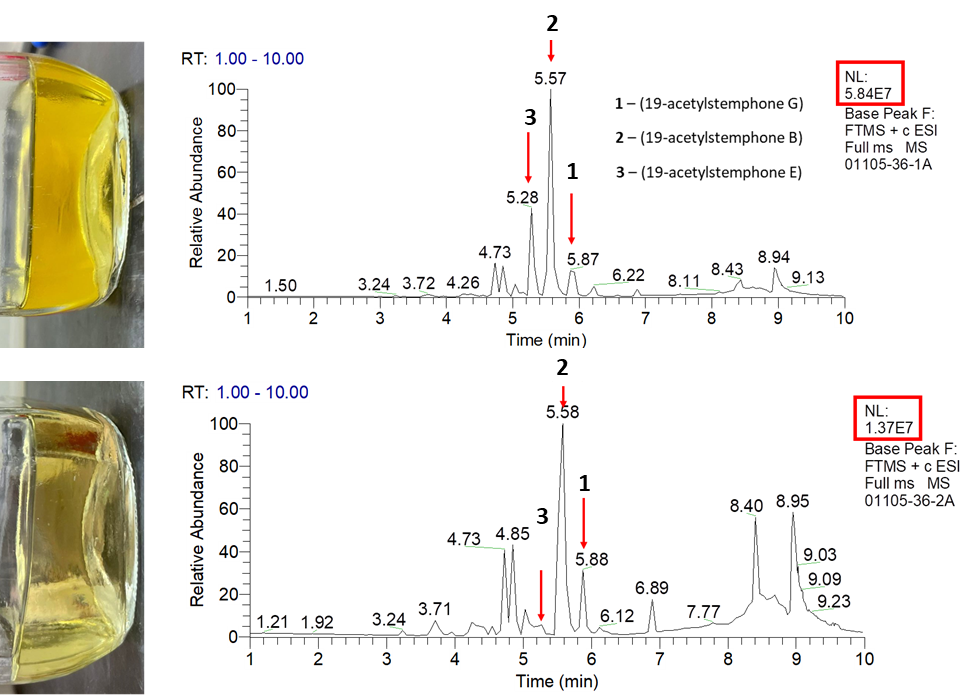


Deionized Water

Nanopure Water


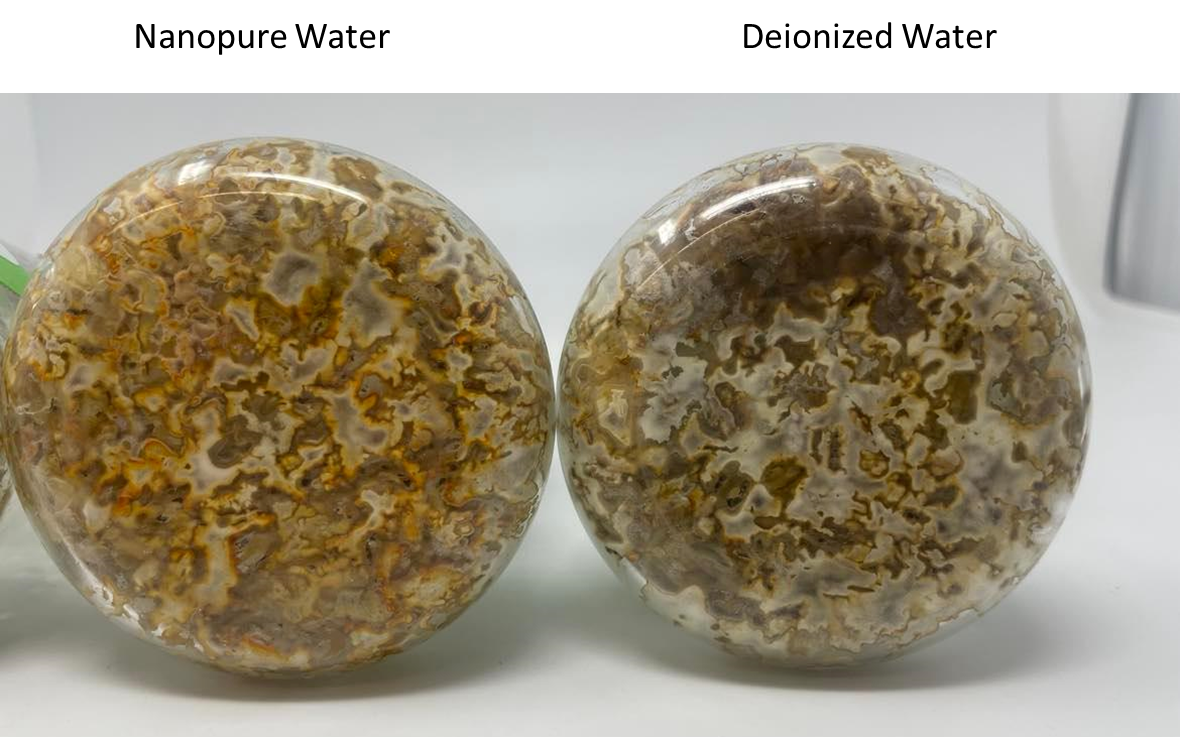


# **Figure S24.** Effect of growth conditions on metabolomic profiles.

The chromatogram on top is from the extract of NRRL 5074 when grown with nanopure water. The chromatogram on the bottom is from the same fungal extract when grown with deionized water. The difference in the color of the extract is also apparent to naked eyes as can be seen on the left side of the figure and in the culture itself as can be seen in the bottom panel of the figure.

**Reference**

Al Subeh, Z.Y., H.A. Raja, S. Monro, L. Flores-Bocanegra, T. El-Elimat, C.J. Pearce, S.A. McFarland, and N.H. Oberlies. 2020. Enhanced Production and Anticancer Properties of Photoactivated Perylenequinones. *Journal of natural products*. 83:2490-2500.

Al Subeh, Z.Y., H.A. Raja, J.C. Obike, C.J. Pearce, M.P. Croatt, and N.H. Oberlies. 2021. Media and strain studies for the scaled production of cis-enone resorcylic acid lactones as feedstocks for semisynthesis. *The Journal of Antibiotics*. 74:496-507.

Caesar, L.K., F.A. Butun, M.T. Robey, N.J. Ayon, R. Gupta, D. Dainko, J.W. Bok, G. Nickles, R.J. Stankey, D. Johnson, D. Mead, K.B. Cank, C.E. Earp, H.A. Raja, N.H. Oberlies, N.P. Keller, and N.L. Kelleher. 2023. Correlative metabologenomics of 110 fungi reveals metabolite–gene cluster pairs. *Nature Chemical Biology*.

Cank, K.B., J.M. Henkin, A.G. Cook, and N.H. Oberlies. 2021. Droplet probe: A non-destructive residue analysis of Wari ceramics from the imperial heartland. *Journal of Archaeological Science*. 134:105468.

Dolomanov, O.V., L.J. Bourhis, R.J. Gildea, J.A.K. Howard, and H. Puschmann. 2009. OLEX2: a complete structure solution, refinement and analysis program. 42:339-341.

El-Elimat, T., M. Figueroa, B.M. Ehrmann, N.B. Cech, C.J. Pearce, and N.H. Oberlies. 2013. High-Resolution MS, MS/MS, and UV Database of Fungal Secondary Metabolites as a Dereplication Protocol for Bioactive Natural Products. *Journal of natural products*. 76:1709-1716.

Graf, T.N., D. Kao, J. Rivera-Chávez, J.M. Gallagher, H.A. Raja, and N.H. Oberlies. 2020. Drug Leads from Endophytic Fungi: Lessons Learned via Scaled Production. *Planta Med*. 86:988-996.

Kvalheim, O.M., H.-y. Chan, I.F.F. Benzie, Y.-t. Szeto, A.H.-c. Tzang, D.K.-w. Mok, and F.-t. Chau. 2011. Chromatographic profiling and multivariate analysis for screening and quantifying the contributions from individual components to the bioactive signature in natural products. *Chemometrics and Intelligent Laboratory Systems*. 107:98-105.

Paguigan, N.D., T. El-Elimat, D. Kao, H.A. Raja, C.J. Pearce, and N.H. Oberlies. 2017. Enhanced dereplication of fungal cultures via use of mass defect filtering. *The Journal of Antibiotics*. 70:553-561.

Pluskal, T., S. Castillo, A. Villar-Briones, and M. Orešič. 2010. MZmine 2: Modular framework for processing, visualizing, and analyzing mass spectrometry-based molecular profile data. *BMC Bioinformatics*. 11:395.

Rigaku. 2022. *CrysAlis* PRO. Oxford Diffraction /Agilent Technologies UK Ltd., Yarnton, England.

Sheldrick, G.M. 2008. A short history of SHELX. *Acta crystallographica. Section A, Foundations of crystallography*. 64:112-122.

Sheldrick, G.M. 2015. SHELXT - integrated space-group and crystal-structure determination. *Acta crystallographica. Section A, Foundations and advances*. 71:3-8.

Sica, V.P., H.A. Raja, T. El-Elimat, V. Kertesz, G.J. Van Berkel, C.J. Pearce, and N.H. Oberlies. 2015. Dereplicating and Spatial Mapping of Secondary Metabolites from Fungal Cultures in Situ. *Journal of natural products*. 78:1926-1936.

Tyler, A.R., R. Ragbirsingh, C.J. McMonagle, P.G. Waddell, S.E. Heaps, J.W. Steed, P. Thaw, M.J. Hall, and M.R. Probert. 2020. Encapsulated Nanodroplet Crystallization of Organic-Soluble Small Molecules. *Chem*. 6:1755-1765.

Vandermolen, K.M., H.A. Raja, T. El-Elimat, and N.H. Oberlies. 2013. Evaluation of culture media for the production of secondary metabolites in a natural products screening program. *AMB Express*. 3:71.

Wang, M., J.J. Carver, V.V. Phelan, L.M. Sanchez, N. Garg, Y. Peng, D.D. Nguyen, J. Watrous, C.A. Kapono, T. Luzzatto-Knaan, C. Porto, A. Bouslimani, A.V. Melnik, M.J. Meehan, W.T. Liu, M. Crüsemann, P.D. Boudreau, E. Esquenazi, M. Sandoval-Calderón, R.D. Kersten, L.A. Pace, R.A. Quinn, K.R. Duncan, C.C. Hsu, D.J. Floros, R.G. Gavilan, K. Kleigrewe, T. Northen, R.J. Dutton, D. Parrot, E.E. Carlson, B. Aigle, C.F. Michelsen, L. Jelsbak, C. Sohlenkamp, P. Pevzner, A. Edlund, J. McLean, J. Piel, B.T. Murphy, L. Gerwick, C.C. Liaw, Y.L. Yang, H.U. Humpf, M. Maansson, R.A. Keyzers, A.C. Sims, A.R. Johnson, A.M. Sidebottom, B.E. Sedio, A. Klitgaard, C.B. Larson, C.A.B. P, D. Torres-Mendoza, D.J. Gonzalez, D.B. Silva, L.M. Marques, D.P. Demarque, E. Pociute, E.C. O'Neill, E. Briand, E.J.N. Helfrich, E.A. Granatosky, E. Glukhov, F. Ryffel, H. Houson, H. Mohimani, J.J. Kharbush, Y. Zeng, J.A. Vorholt, K.L. Kurita, P. Charusanti, K.L. McPhail, K.F. Nielsen, L. Vuong, M. Elfeki, M.F. Traxler, N. Engene, N. Koyama, O.B. Vining, R. Baric, R.R. Silva, S.J. Mascuch, S. Tomasi, S. Jenkins, V. Macherla, T. Hoffman, V. Agarwal, P.G. Williams, J. Dai, R. Neupane, J. Gurr, A.M.C. Rodríguez, A. Lamsa, C. Zhang, K. Dorrestein, B.M. Duggan, J. Almaliti, P.M. Allard, P. Phapale, et al. 2016. Sharing and community curation of mass spectrometry data with Global Natural Products Social Molecular Networking. *Nature biotechnology*. 34:828-837.
